# Sphingosine 1-phosphate signalling contributes to transforming growth factor-β1-induced myofibroblast differentiation in oral cancer

**DOI:** 10.7717/peerj.21673

**Published:** 2026-09-07

**Authors:** Kathyleen Hui Lin Chong, Wee Lin Wong, Deron R. Herr, Wai-Leng Lee, Lee Fah Yap, Ian C. Paterson

**Affiliations:** 1 Department of Oral and Craniofacial Sciences, Universiti Malaya, Kuala Lumpur, Malaysia; 2 School of Medicine, Indiana University, Terre Haute, Indiana, United States; 3 School of Science, Monash University, Kuala Lumpur, Malaysia; 4 Oral Cancer Research & Coordinating Centre, Universiti Malaya, Kuala Lumpur, Malaysia

**Keywords:** Myofibroblast, Sphingosine 1-phosphate, Oral cancer, TGF-beta, Fibroblast, CAF

## Abstract

The prognosis for patients with oral squamous cell carcinoma (OSCC) is poor and novel ways to manage the disease are required. The density of myofibroblastic cancer-associated fibroblasts (myoCAFs) within the OSCC stroma correlates with poor prognosis, suggesting that myoCAFs could be targetted therapeutically. OSCC cells have been shown to regulate myoCAF differentiation *via* mechanisms involving transforming growth factor-β1 (TGF-β1), but the further downstream mechanisms are yet to be clearly defined. In this study, we aimed to examine the involvement of sphingosine 1-phosphate (S1P) signalling in mediating myofibroblast differentiation. Treatment of normal oral fibroblasts with OSCC conditioned media (CM) or TGF-β1 induced the expression of α-smooth muscle actin (α-SMA), a well-recognised marker of fibroblast activation/myofibroblasts. TGF-β1 up-regulated expression of sphingosine kinase 1 (SK1), but not sphingosine kinase 2 (SK2). However, inhibiting either of the SKs abrogated the induction of α-SMA expression by TGF-β1 and OSCC CM. The effect of inhibiting SK1/2 could not be rescued by exogenous S1P suggesting a possible role for intracellular S1P. Normal oral fibroblasts were shown to express predominantly sphingosine 1-phosphate receptor 2 (S1PR2) and sphingosine 1-phosphate receptor 3 (S1PR3) receptors. The induction of α-SMA expression by OSCC CM was abrogated with a S1PR2 antagonist, whilst α-SMA expression was upregulated following treatment with a S1PR2 agonist. Using pharmacological modulation of α-SMA expression, our data show that SKs and S1PR2 contribute to myofibroblast differentiation in OSCC. These results highlight the potential of inhibiting S1P signalling to inhibit myoCAF development.

## Introduction

Oral cancer (lip, tongue and mouth) is a major world health issue that is particularly prevalent in India and South East Asia. Approximately 90% of cases are diagnosed as oral squamous cell carcinoma (OSCC) and more than 350,000 new cases are diagnosed each year. Patient prognosis is poor with a 5-year survival rate of approximately 50% (Miranda-Filho et al., 2020). Treatment often comprises surgery, chemotherapy and radiotherapy, but loco-regional recurrences, distant metastases and second primary tumours occur frequently and are responsible for the poor patient prognosis (Miranda-Filho et al., 2020). Whilst our understanding of the molecular basis for the development of OSCC is comprehensive (Leemans, Snijders & Brakenhoff, 2018), molecular targeted therapies are not in routine use, and innovative ways to manage the disease are needed.

It is now well recognised that the tumour microenvironment (TME) influences tumour progression and therapeutic responses. Among the many cell types within the TME, cancer-associated fibroblasts (CAFs) have been shown to contribute to many of the hallmarks of cancer (Hanahan & Coussens, 2012). A number of different CAF phenotypes have been reported and this heterogeneity is dependent on the cell type and tumour of origin. These CAF subtypes include myofibroblastic CAFs (myoCAFs), inflammatory CAFs (iCAFs) and antigen-presenting CAFs (apCAFs), plus a number of less well defined subtypes (Sahai et al., 2020; Cords et al., 2023). In many solid tumours, including OSCC, CAFs myoCAFs are the predominant subtype. These cells display a myofibroblast-like phenotype similar to myofibroblasts in healing wounds in that they express α-smooth muscle actin (α-SMA) and secrete a variety of collagen subtypes (Kalluri, 2016). Notably, the density of these myoCAFs within the TME of OSCCs is associated with poor patient outcomes (Marsh et al., 2011; Dourado et al., 2018), leading to the suggestion that myoCAFs could be targeted in a therapeutic context (Sahai et al., 2020). MyoCAFs are derived from resident normal fibroblasts that are induced to differentiate into a myofibroblast phenotype by transforming growth factor-β1 (TGF-β1) (Hassona et al., 2013), although the molecular mechanisms downstream of TGF-β1 are not clear. A deeper understanding of the mechanisms that regulate myoCAF formation is needed if we are to exploit these cells as therapeutic targets.

Sphingosine-1-phosphate (S1P) is a bioactive lipid generated by the phosphorylation of sphingosine by two sphingosine kinases (SKs), SK1 and SK2 (Pyne, Adams & Pyne, 2016). S1P is involved in a wide variety of cellular processes, including proliferation and apoptosis and deregulated S1P signalling is associated with diseases such as cancer and fibrosis (Kano, Aoki & Hla, 2022; Pyne, Adams & Pyne, 2016). These effects are largely due to the binding to one or more of a family of five G-protein coupled receptors, termed S1PR1–5, which then stimulate multiple signalling cascades (Patmanathan et al., 2017). However, some intracellular effects of S1P have been reported and this are thought to predominantly be mediated by SK2-derived S1P (Hait et al., 2009), although intracellular effects of SK1 have also been reported (Kohno et al., 2006).

It is well known that S1P signalling regulates many aspects of tissue fibrosis, including the differentiation of myoblasts and normal fibroblasts into myofibroblasts (Donati et al., 2021). The actions of S1P in the pathogenesis of tissue fibrosis has been shown in some cases to act downstream of TGF-β1 (Donati et al., 2021). For example, SK1 mediates the transdifferentiation of myoblasts to myofibroblasts induced by TGF-β1 (Cencetti et al., 2010) and S1P was found to mediate the profibrotic action of TGF-β1 in an *in vitro* model of uterine adenocarcinoma cells (Bernacchioni et al., 2021). The role of S1P signalling in the development and function of CAFs, and myoCAFS in particular, is much less clear. However, in melanoma, SK1 produces S1P in melanoma cells, which subsequently promotes the differentiation of fibroblasts to myofibroblasts (Albinet et al., 2014) and SK1 was shown to be required for TGF-β mediated differentiation of normal fibroblasts to myofibroblasts in ovarian cancer (Beach et al., 2016). Therefore, the involvement of the S1P signalling pathway in mediating myofibroblast differentiation induced by OSCC cells, which occurs *via* the action of TGF-β1, warrants investigation.

In this study, we aimed to examine the involvement of sphingosine 1-phosphate (S1P) signalling in mediating myofibroblast differentiation. We used the induction of α-SMA expression as a marker of myofibroblast differentiation and the development of the myoCAF phenotype, as α-SMA is invariably expressed by myoCAFs and the up-regulation of α-SMA expression has been described in multiple studies involving oral fibroblasts (Hassona et al., 2013; Melling et al., 2018; Mellone et al., 2022). Using pharmacological antagonists/agonists of SKs and S1PR2/3, we show that SKs and S1PRs, predominantly S1PR2, contribute to the development of the myoCAF phenotype in response TGF-β1 and OSCC cell conditioned media (CM). Taken together, our data show that inhibitors of sphingosine kinases and/or S1PR2 might be used to to inhibit myoCAF development.

## Materials and Methods

### Materials

Sphingosine-1-phosphate (S1P; D-erythro S1P; Avanti Polar Lipids, Alabaster, AL, USA) was prepared as described previously (Patmanathan et al., 2016). Specifically, S1P was dissolved in 95% methanol, dried under a stream of nitrogen gas and stored at −20 °C in aliquots of 100 nmol. Prior to use, S1P was constituted in 4 mg/mL fatty acid-free human albumin (FAFA; Sigma, St. Louis, MO, USA) at a final concentration of 200 µM.

Cisplatin [cis-Diammineplatinum(II) dichloride] and JTE013 [1-[1,3-Dimethyl-4-(2-methylethyl)-1H-pyrazolo[3,4-b] pyridin-6-yl]-4-(3,5-dichloro-4-pyridinyl)-semicarbazide] were obtained from Tocris Biosciences (Bristol, UK). FTY720 (2-Amino-2-(2-(4-octylphenyl)ethyl)propane-1,3-diol, HCl). SK1 inhibitor, PF543 and SK2 inhibitor, ABC294640 were both obtained from Selleck (Shanghai, China). Recombinant human TGF-β1 was from Miltenyi Biotec (Bergisch Gladbach, North Rhine-Westphalia, Germany) and the specific inhibitor for TGF-β type 1 receptor, SB-431542 was from Selleck (Shanghai, China). A specific inhibitor of S1PR2, JTE013 was obtained from Tocris Biosciences (Bristol, UK), while specific S1P receptor agonist CYM5520 and CYM5541 were obtained from Sigma–Aldrich (St. Louis, MO, USA).

### Cell lines and culture

Normal human oral fibroblasts (NHOFs) (NHOF1, NHOF2, NHOF7) were kind gifts from Professor EK Parkinson (Queen Mary, University of London). The fibroblasts were derived from explant cultures of normal oral mucosa as described previously (James et al., 2015). The NHOFs were from different donors and therefore represent biological replicates. The OSCC cell lines (H103, H376, H400 and H357) used have also been described previously (Prime et al., 1990).

### Collection of conditioned media

OSCC cells were cultured 75 cm^2^ cell culture flasks until 80–90% confluent. The culture media were removed and the cells washed twice with PBS. The cells were then further incubated in 10 ml of serum-free medium 48 h before collection. The conditioned media (CM) were collected and then centrifuged in 4,000 rpm for 20 min to remove cell debris. The remaining cells were trypsinised and counted to obtain the number of cells per flask and the volume of CM used for experiments was normalised to 3 × 10^5^/mL. The CM was stored in −80 °C prior to use.

### Western blotting analysis

Cells were lysed with ice-cold NP40 lysis buffer supplemented with Protease Inhibitor Cocktail Set III (Calbiochem, Germany) and Halt Phosphatase Inhibitor Cocktail (Thermo Scientific, Waltham, MA, USA). Western blotting was carried out using standard protocols. Antibodies against α-SMA were purchased from Dako (M0851; 1:2,500; Santa Clara, CA, USA) whilst antibodies against GAPDH (ab9485; 1:5,000) were obtained from Abcam (Cambridge, UK). Blocking and antibody incubations were performed in 5% non-fat milk in Tris-buffered Saline (TBS) 150 nM NaCL, 50 mM Tris-HCl (pH 7.6) with 0.1% Tween-20 (TBST). Secondary antibodies (1:5,000) used in this study were HRP-conjugated goat anti-rabbit IgG or goat anti-mouse IgG (1:5,000; Sigma–Aldrich, St. Louis, MO, USA). Bound antibodies were detected as described previously (Patmanathan et al., 2016). Briefly, bound antibodies were detected with WesternBright Sirius enhanced chemiluminescence (ECL) reagent (Advansta, San Jose, CA, USA) using an Odyssey FC Imaging System (LI-COR Biosciences, Lincoln, NE, USA). Densitometry analyses were conducted using ImageJ software. Densitometric values were normalised to GAPDH and expressed relative to untreated fibroblasts (set to = 1). The densitometric value is calculated based on the actual blot shown in the Figures. To show the effects of CYM5520 and CYM5541, data were combined from three independent experiments and shown in the supplementary figures.

### Quantitative real-time polymerase chain reaction

Total RNA was extracted from cells using an NucleoSpin^®^ RNA II kit (Macherey-Nagel, Düren, Germany) and cDNA generated using a High-Capacity cDNA Reverse Transcription kit (Applied Biosystems, Life Technologies, Carlsbad, CA, USA), following the manufacturers’ instructions. Quantitative real-time polymerase chain reaction (QPCR) was performed in triplicate using the 7500 Fast Real-Time PCR system (Applied Biosystems, Carlsbad, CA, USA) and TaqMan^®^ Gene Expression Assays (SPHK1: Hs00184211_m1; S1PR1: Hs001922614_s1; S1PR2: A139R4J; S1PR3: Hs00245464_s1; S1PR4: Hs02330084_s1; S1PR5: Hs00928195_s1) (Applied Biosystems, Carlsbad, CA, USA). GAPDH (4326317E) (Applied Biosystem, Carlsbad, CA, USA) was amplified in the same reaction to serve as an internal control for normalization. The qPCR results were analysed using 7500 Software v2.0 (Applied Biosystems, Carlsbad, CA, USA) and fold changes in gene expression were determined using the comparative threshold cycle method (ΔΔCt).

### Statistical analyses

All statistical analyses were performed using GraphPad Prism Version 5 (GraphPad Software Inc., Boston, MA, USA). Statistical differences between experimental groups were evaluated by one-way analysis of variance (ANOVA) or Student’s t-test. *p*-values < 0.05 were regarded as significant.

## Results

### Conditioned media from OSCC cells induces α-SMA expression in normal human oral fibroblasts *via* the TGF-β signalling pathway

**Figure 1:**
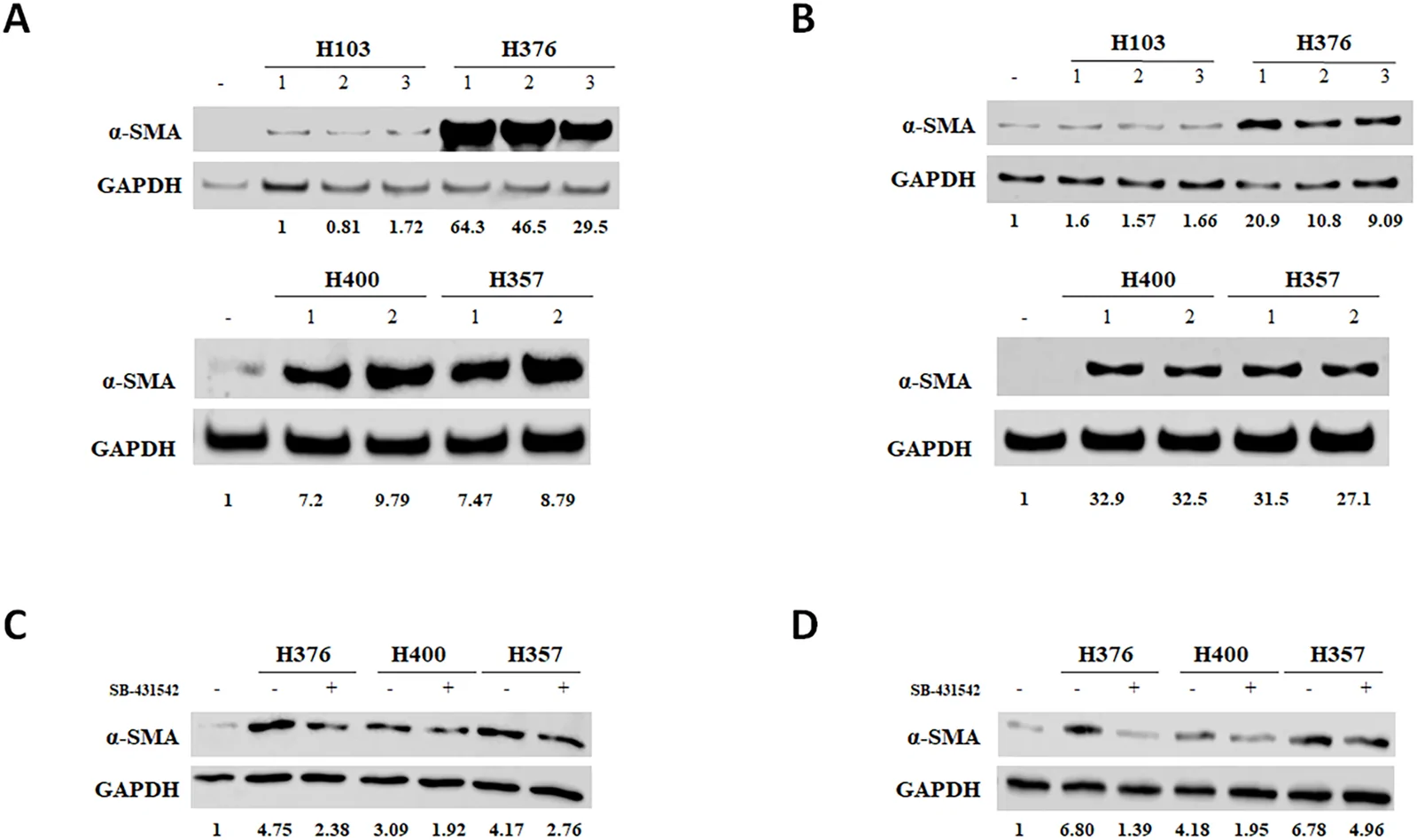
OSCC CM upregulates α-SMA expression in normal oral fibroblasts. CM from three OSCC cell lines H376, H357 and H400, but not H103, induced a-SMA expression in NHOF1 (A) and NHOF2 (B), as determined by Western blotting. The results shown are from two (H357, H400) or three (H103, H376) independent samples of CM compared to control (−). This effect was blocked with a TGF-β type I receptor inhibitor, SB 431542, in both NHOF1 (C) and NHOF2 (D). The intensity of the bands are normalized to GAPDH and expressed relative to the cells treated with vehicle control (=1). Similar results were obtained in three independent experiments.

We first examined whether CM from OSCC-derived cell lines induced α-SMA expression in NHOFs. CM from H357, H376 and H400, but not H103, caused an up-regulation of α-SMA protein levels in two cultures of normal oral fibroblasts, NHOF 1 (Fig. 1A) and NHOF 2 (Fig. 1B). To confirm that TGF-β mediated the effects of OSCC CM on normal fibroblasts, NHOF1 and NHOF2 cells were treated with CM from H376, H400 and H357 cells in the presence the TGF-β type 1 receptor inhibitor, SB-431542. A reduction in OSCC CM-induced α-SMA protein expression was observed in both NHOF1 (Fig. 1C) and NHOF2 (Fig. 1D) cells treated with SB-43152. The results show that the majority of OSCC cells can induce myofibroblast differentiation *via* mechanisms that involve TGF-β signalling.

### TGF-β1-induced α-SMA expression is mediated by sphingosine kinases

**Figure 2:**
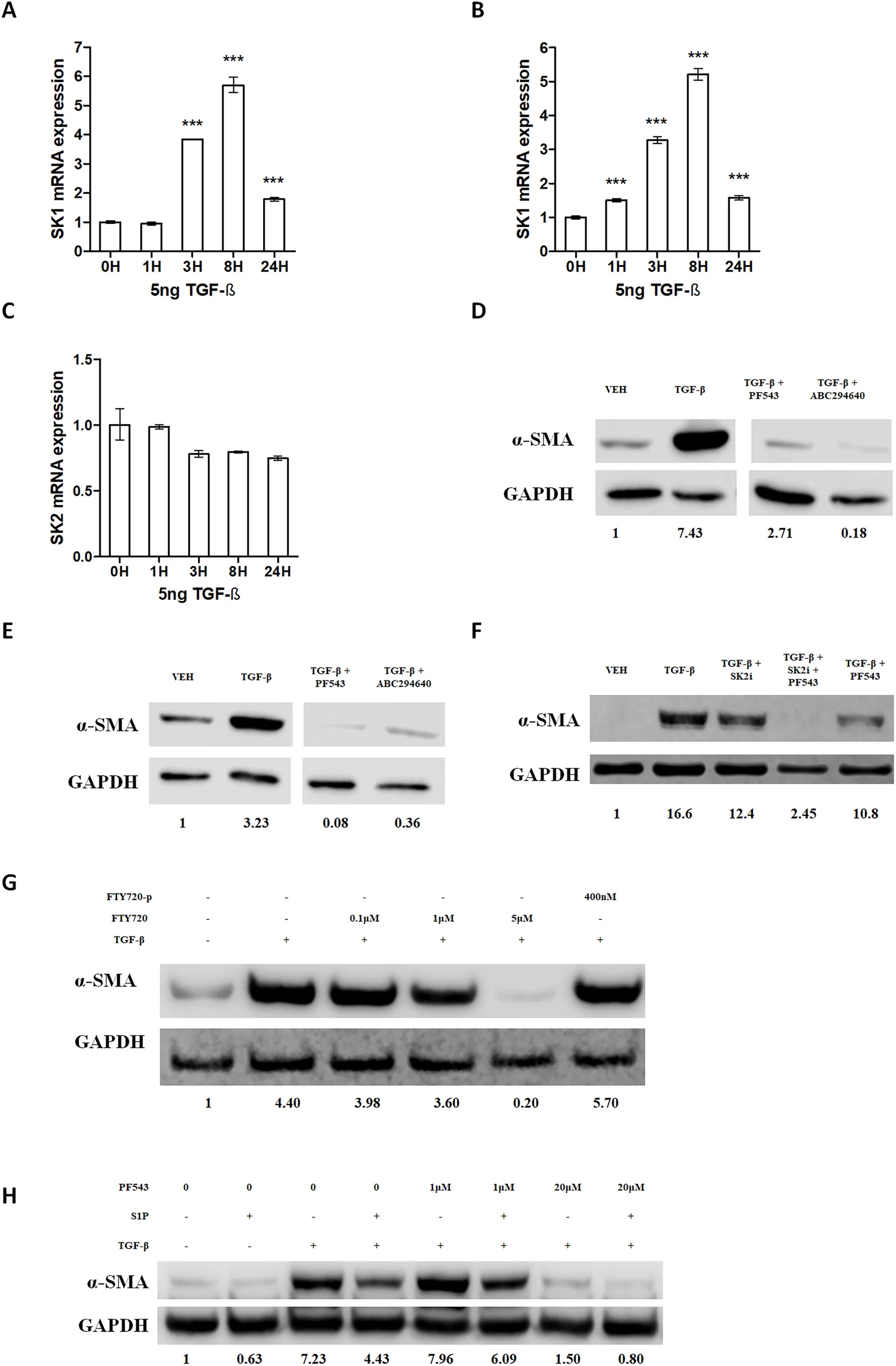
SK activity was required for myofibroblast differentiation induced by TGF-β1. QPCR analysis of the expression of SK1 in NHOF1 (A) and NHOF2 (B) after treatment with TGF-β1 (5 ng/mL). The expression of SK2 was also examined after TGF-β1 treatment (5 ng/mL) in NHOF7 (C). The expression of SK1/2 was normalised to GAPDH and are expressed as the relative expression between the cells treated with TGF-β and their respective controls (normalized to 1). ***p < 0.001, one-way (ANOVA). Western blotting images showing the downregulation of α-SMA protein levels in (D) NHOF1 and (E) NHOF2 following treatment with competitive SK1 inhibitor, PF543 (20 μM) and selective SK2 inhibitor, ABC294640 (25 μM). The intensity of the bands are normalized to GAPDH and expressed relative to the cells treated with vehicle control (=1). The experiments were repeated twice with similar results. Similar effects were seen with the SK2 inhibitor SK2i and this was greater when combined with the SK1 inhibitor, PF543 (F). In NHOF2 cells, the S1PR antagonist, FTY720, but not FTY720-P in NHOF2 cells reduced α-SMA protein expression (G). Similar results were obtained using NHOF1 (Fig. S1). Exogenous S1P did not rescue the effects of SK inhibition in NHOF2 (H) and also in NHOF1 (Fig. S2).

We next aimed to investigate a possible role for S1P signalling in the development of MyoCAFs. We first examined *SK1* and *SK2* mRNA expression in NHOFs treated with TGF-β1 by QPCR. *SK1* mRNA expression increased in a time-dependent manner (0–8 h), increasing approximately 5-fold after 8 h, before returning close to baseline after 24 h (Figs. 2A and 2B), whilst SK2 mRNA levels were not increased (Fig. 2C). These results suggest that TGF-β1 might induce fibroblast activation through the S1P signalling pathway. Therefore, we next used chemical inhibitors of SK1 and SK2 to investigate the involvement of these kinases in this effect. Two SK1 inhibitors (FTY720 and PF543) and one SK2 inhibitor (ABC294640) were used. FTY720 is a S1P analogue and acts as a competitive inhibitor of SK1, while PF543 is a selective competitive SK1 inhibitor with a *K*_i_ of 3.6 nM (Lim et al., 2012; Schnute et al., 2012). ABC294640 competes with sphingosine with a *K*_i_ of 9.8 μM (French et al., 2010). TGF-β1 upregulated α-SMA in NHOF1 and NHOF2 and this effect was attenuated by PF543 and ABC294640 (Figs. 2D and 2E). In support of these observations, similar results were found using the SK2 inhibitor, SK2i and inhibiting SK1 and SK2 together had a greater effect than inhibiting either SK alone in NHOF2 (Fig. 2F). Furthermore, FTY720 also inhibited α-SMA expression in response to TGF-β1, but the phosphorylated form of FTY720 (FTY720-p), which acts as a S1P receptor agonist at low concentrations, had no effect (Fig. 2G; Fig. S1). These results strongly suggest that SK activity downstream of TGF-β1 mediates myofibroblast differentiation.

### Exogenous S1P did not rescue the effect of SK inhibition

As inhibition of SK activity mediated fibroblast activation in response to TGF-β1, the possibility that exogenous S1P treatment could rescue the down-regulation of α-SMA expression caused by SK1 inhibition was investigated. NHOF2 cells were treated with the SK1 inhibitor, PF-543, together with exogenous S1P and the results showed that S1P alone did not increase α-SMA levels and S1P did not enhance the up-regulation of α-SMA by TGF-β1 (Fig. 2H). To further confirm that exogenous S1P has no effect on α-SMA levels in normal fibroblasts, cells were treated with PF-543 and S1P in the presence of TGF-β1. The α-SMA levels in normal fibroblasts remained unchanged with or without treatment of S1P after SK1 inhibition (Fig. 2H). Similar results were obtained using NHOF 1 cells (Fig. S2). The results showed that exogenous S1P did not affect α-SMA levels in normal fibroblast in a similar way as TGF-β1 and S1P could not rescue the in inhibition of SK1 by PF543. The results demonstrate that exogenous S1P does not directly induce myofibroblast differentiation, which suggests the possible involvement of endogenous S1P.

### SK inhibition abrogates α-SMA-induced by OSCC CM in normal fibroblast

**Figure 3:**
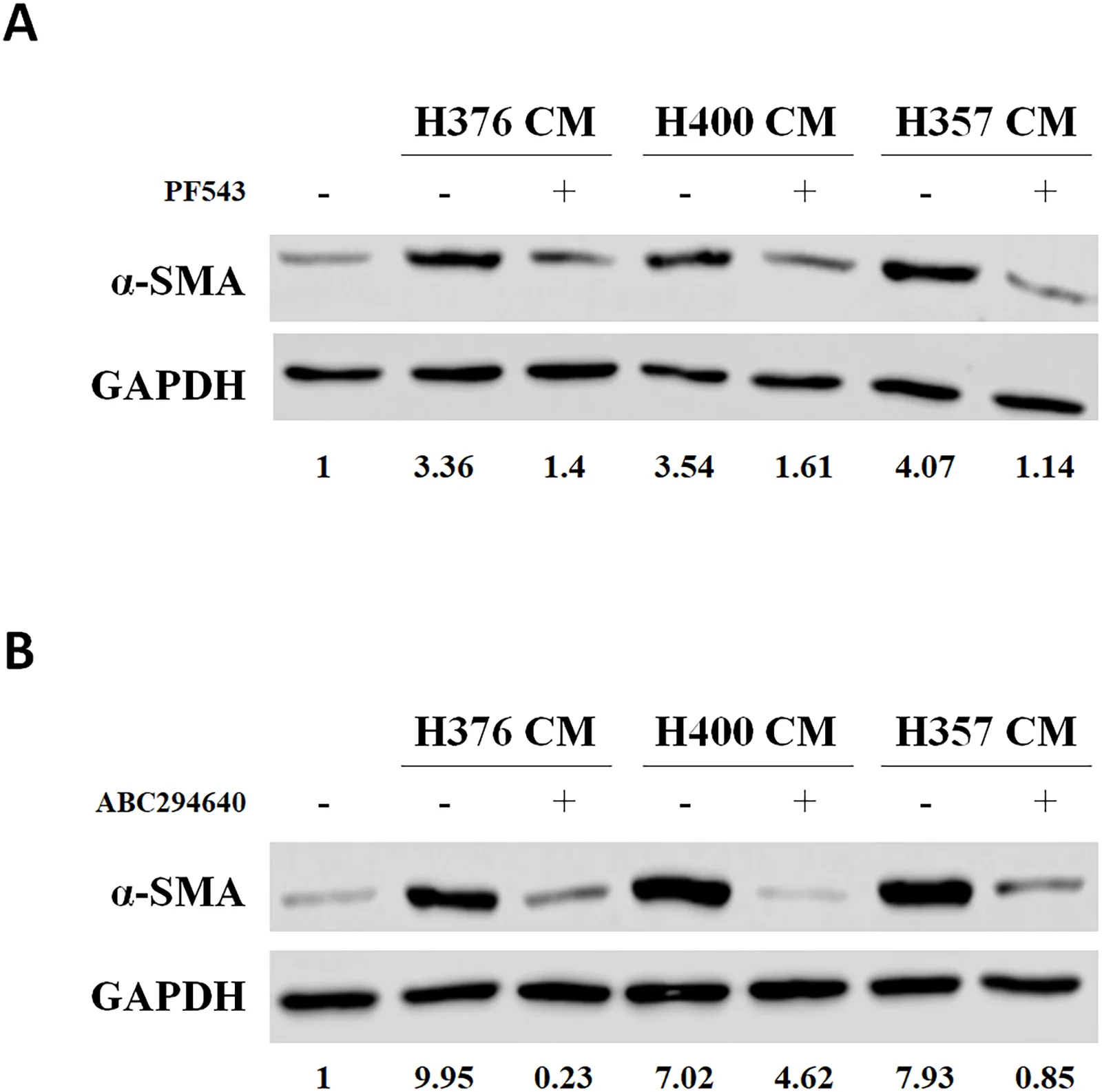
α-SMA expression induced by OSCC CM was inhibited by SK inhibitors. CM-induced α-SMA expression in NHOF1 was inhibited after treatment with the SK1 inhibitor, PF543 (20 μM), for 72 h (A), as determined by Western blotting. Similarly, treatment of NHOF1 with the SK2 inhibitor, ABC29460 (25 μM), for 72 h inhibited OSCC CM-induced α-SMA expression (B). The intensity of the bands are normalized to GAPDH and expressed relative to control treated cells (=1). Similar results were obtained in three independent experiments.

The results of the present study showed that SKs mediate the development of a myofibroblastic phenotype in response to TGF-β1, so we next examined whether SKs are also involved in the induction of α-SMA expression by OSCC cells. The SK1 inhibitor, PF543 attenuated the increase in α-SMA protein expression in OSCC CM treated NHOF2 (Fig. 3A). Similarly, α-SMA protein levels induced by OSCC CM were downregulated by the SK2 inhibitor, ABC292460 (Fig. 3B).

### Myofibroblast differentiation involves S1PR2

**Figure 4:**
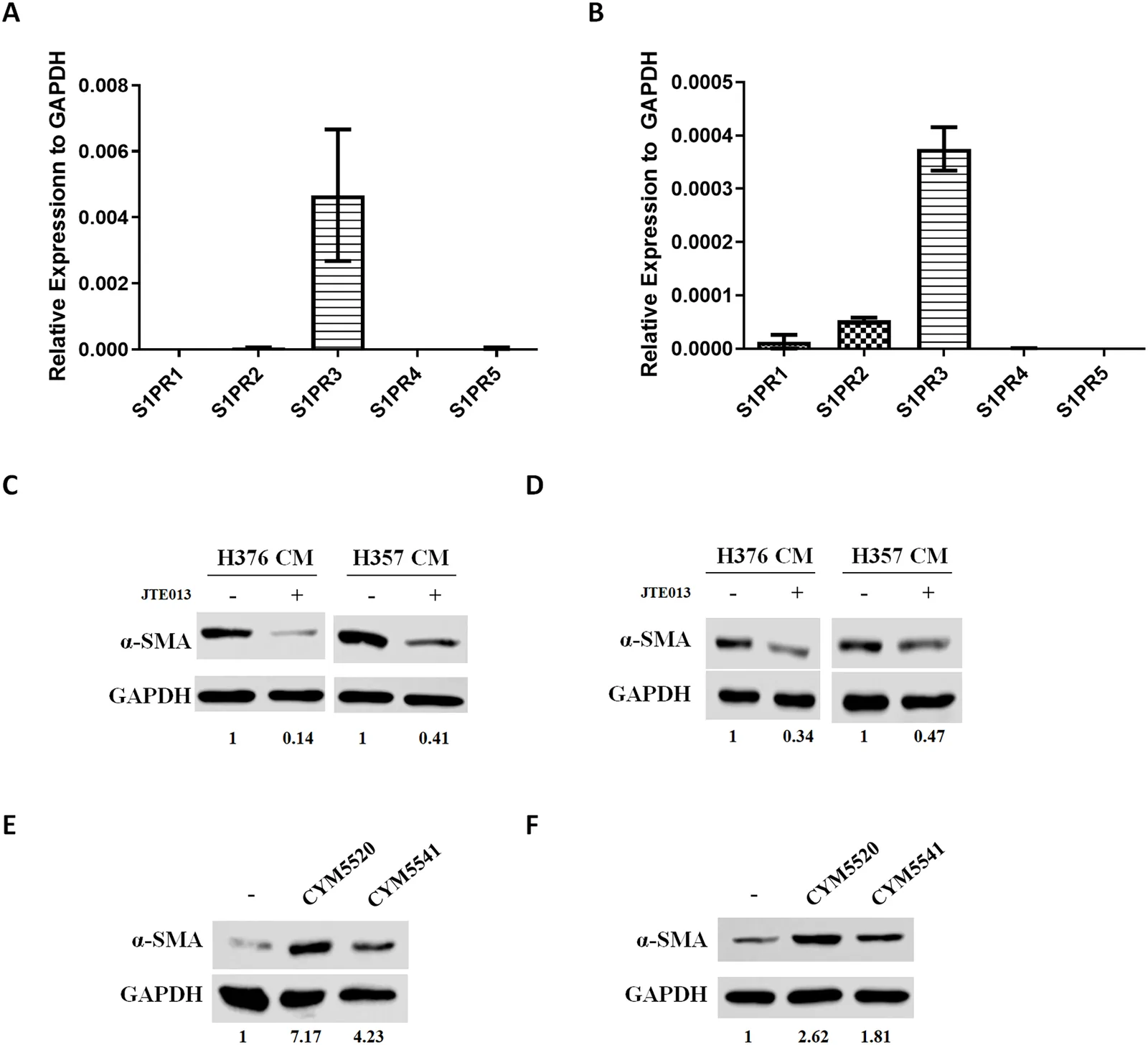
S1PR2 is involved in myofibroblast differentiation. QPCR analysis showed that NHOF1 (A) NHOF2 (B) expressed both S1PR2 and S1PR3, whilst S1P4 was undetectable. S1PR1 was expressed only in NHOF2 and S1PR5 was expressed only by NHOF1. S1PR expression was normalised to GAPDH and are expressed as mean ± SD of triplicates. The data presented are representative of three independent experiments. Treatment of NHOF1 (C) and NHOF2 (D) with S1PR2 antagonist, JTE013 (5 μM) for 72 h inhibited OSCC CM induced-α-SMA protein levels, as shown by Western blotting. This effect was shown to be significant (Fig. S4) The selective S1PR2 allosteric agonist, CYM5520 (10 μM), strongly induced α-SMA levels in both NHOF1 (E) and NHOF2 (F). A similar effect was observed with the selective S1PR3 allosteric agonist, CYM5541 (10 μM), but to a lesser extent The intensity of the bands are normalized to GAPDH and expressed relative to the cells treated with vehicle control (=1).

The results of this study have demonstrated the involvement of sphingosine kinases, the enzymes that produce S1P, in fibroblast activation. Therefore, we further investigated the S1P signalling pathway by examining the possible involvement of S1P receptors in this process. First, we examined the expression of S1P receptors in NHOFs. Our previously published RNAseq data (Lai et al., 2019) indicated that NHOF1 and NHOF2 expressed high levels of S1PR2 and S1PR3. Both fibroblast strains had low levels of S1PR1 RNA expression while the levels of S1PR4 and S1PR5 were very low (Fig. S3). These results were validated by QPCR, which showed that S1PR2 and S1PR3 mRNA were readily detected in both cell lines, whilst S1PR1 was also expressed but at a lower level (Figs. 4A and 4B). S1PR4 was not detected in either cell line and S1PR5 was only detected in NHOF1 (Figs. 4A and 4B).

We next examined the involvement of S1PR2 and S1PR3 in myofibroblast differentiation induced by OSCC CM. To investigate the potential involvement of the S1PR2 receptor, S1PR2 activity was inhibited using a competitive S1PR2 antagonist, JTE013 (Long et al., 2010), in NHOF cells. The results showed that JTE013 downregulated α-SMA expression in both NHOF1 (Fig. 4C) and NHOF2 (Fig. 4D) cells treated with CM from H376 and H357 OSCC cells. The effects of JTE013 were statistically significant as shown in Fig. S4. By contrast, the S1PR1/3 antagonist, VPC23019 (Davis et al., 2005), appeared to have a lesser effect on α-SMA levels (Fig. S2). To further investigate the role of S1PR2/3 in fibroblast activation, selective agonists for S1PR2 (CYM5520; Satsu et al., 2013) and S1PR3 (CYM5541; Jo et al., 2012) were used. Treatment of NHOFs with CYM5520 induced strong α-SMA protein expression, whilst CYM5541 had a lesser effect on α-SMA expression in both NHOF1 (Fig. 4E) and NHOF2 (Fig. 4F), although the results from three combined independent experiments did not reach statistical significance (Fig. S5).

## Discussion

MyoCAFs have been described in many different solid tumour types and play a role in tumour progression and resistance to therapy, including immunotherapies. In OSCC, a high density of myoCAFs correlates with poor patient survival, so understanding the mechanisms of myoCAF differentiation is needed in order to counteract myoCAF functions therapeutically. Myofibroblast differentiation in OSCC was shown to be induced by TGF-β1 by mechanisms that include an increase in reactive oxygen species (ROS) (Hassona et al., 2013). These findings were extended recently by Mellone et al. (2022), who showed that this effect was mediated by TGF-β1-dependent activation of ATM by the ROS-producing enzyme NOX4. Other signalling pathways are likely to be involved, however, because previous studies have found components of the S1P signalling pathway to be crucial in regulating TGF-β-induced myofibroblast differentiation (Cencetti et al., 2010; Kono et al., 2007; Beach et al., 2016). We therefore examined S1P and its receptors in myoCAF differentiation in oral cancer in more detail.

In the present study, we confirmed the involvement of TGF-β1 in myofibroblast differentiation by showing that CM from three out of four OSCC cell lines induced α-SMA expression in NHOFs and this effect was blocked with a type I TGF-β receptor antagonist. It is not clear why CM from H103 had no effect on α-SMA expression. It was reported previously that H103 secreted TGF-β1 into CM *in vitro*, but it is not known what proportion of the TGF-β1 is latent *vs*. active ligand (Fahey et al., 1996). It is possible that amount of active TGF-β1, and possibly other cytokines, is insufficient in CM from H103 cells to elicit an effect on α-SMA expression *in vitro*. Further, treatment of NHOFs with TGF-β1 increased the expression of SK1, which agrees with a previous report (Beach et al., 2016), whilst we did not observe a similar effect on SK2 expression. We subsequently showed that SK activity was required for the induction of TGF-β-induced α-SMA expression in NHOFs, as inhibition of either SK1 or SK2 abolished the effects of both TGF-β1 and CM in NHOFs. Inhibition of both SK1 and SK2 together appeared to have a greater effect, although it is difficult to assess the precise contribution of each SK, as the inhibitors could have some cross-reactivity. The mechanism of SK action in myofibroblast differentiation is unclear because only SK1 and not SK2 transcription was upregulated by TGF-β1. This might suggest an intracellular role for S1P, as SK2 generated S1P is thought to act more intracellularly than SK1-derived S1P, although this difference is not absolute. In support of this hypothesis, exogenous S1P did not up-regulate the expression of α-SMA in NHOFs, nor did it rescue the effects of SK inhibition. Similarly, FTY720-p which acts as a non-selective agonist of the S1PRs (except for S1PR2) also did not induce α-SMA expression. Results from other studies are contradictory because S1P induced fibrotic markers in various cellular contexts, including lung and ovarian fibroblasts (Beach et al., 2016; Urata et al., 2005), but Cencetti et al. (2010) demonstrated that exogenous S1P failed to direct the induction of myofibroblasts. The effect of S1P is likely to be cell and context dependent. Taken together, our data clearly show that SKs are required for myofibroblast differentiation in response to TGF-β1 and/or CM from OSCC cells in oral fibroblasts. Future experiments using gene knockout technologies will be required to confirm the precise roles of SK1 and SK2 in mediating this effect. How SK activity might interact with other pathways such as ATM signalling is not clear, but it has been reported that S1P could attenuate genotoxic-stress-induced DNA damage in sperm and this could be reversed by ATM inhibition (Stobezki et al., 2020). How S1P and ATM signalling might cooperate to regulate myoCAF differentiation, therefore, is worth exploring.

Having shown that normal fibroblasts predominantly express S1PR2 and S1PR3 in the present study, we next examined the role of these receptors in myofibroblast differentiation, using pharmacological inhibitors. The S1PR2 antagonist, JTE013, completely attenuated the upregulation of α-SMA expression induced by OSCC CM, while the S1PR1/3 antagonist, VPC23019, had little or no effect on α-SMA expression. It is cautionary to note that these inhibitors are not totally efficacious or specific. For example, VPC23019 has relatively weak S1PR3 antagonist activity (Salomone & Waeber, 2011) and the selectivity of JTE013 is not absolute, demonstrating some antagonism for S1PR4 (Long et al., 2010). However, the lack of expression of S1PR4 by NHOFs implies that the effects of JTE013 in these cells are specific to S1PR2 and indicate that myofibroblast differentiation is regulated by S1PR2. To confirm the contributions of S1PR2 and/or S1PR3, we investigated the effect of specific agonists. Stimulation of S1PR2 with the agonist, CYM5520, increased strong α-SMA levels in fibroblasts, an effect that was less pronounced using the S1PR3 agonist, CYM5541. Our results of the present study support previous work that showed that the S1PR2 receptor mediates myogenic differentiation (Donati et al., 2005), although S1PR3 has also been implicated in this process (Cencetti et al., 2010), and in myofibroblast differentiation in response to TGF-β1 in ovarian cancer (Beach et al., 2016). It is not clear what the mechanism of S1PR2 activation is in our study because exogenous S1P did not induce α-SMA in NHOFs. However, alternative ways to activate this receptor have been described, as it has been reported that S1PR2 shed *via* breast cancer cell exosomes becomes activated following fusion with fibroblasts to induce ERK activation and proliferation in these cells (El Buri et al., 2018).

MyoCAFs are recognized as being bone fide therapeutic targets for solid tumours and such approaches can be directed at not only inhibiting myoCAF development and function, but also to reverse the myoCAF phenotype to normalize the tumour stroma (Sahai et al., 2020; Mellone et al., 2022). Our results suggest a potential role for SK and S1PR2 inhibitors in this context. It is noteworthy that SKs have been shown to have oncogenic functions and S1PR expression is deregulated in various tumours, alterations that can be targetted therapeutically (Rufail, Bassi & Giussani, 2025; Patmanathan et al., 2017). We have shown previously in OSCC that SK1 and S1PR2 are over-expressed and S1PR2 mediates S1P-induced migration (Patmanathan et al., 2016), which leads to the intriguing possibility that inhibiting S1P signalling may target both tumour cells and myoCAFs in OSCC.

## Conclusions

MyoCAFs are known to contribute to tumour progression and resistance to therapy in many solid tumour types. In OSCC, the density of such myoCAFs correlates with poor patient prognosis, so understanding the mechanisms underlying the development of the myofibroblastic phenotype could lead to new therapies to target CAFs. In this study, we have investigated the role of the S1P signalling pathway in regulating myofibroblast differentiation in OSCC. We show that CM from OSCC cells acting in part *via* TGF-β1 stimulates myofibroblast differentiation, as indicated by the up-regulation of α-SMA expression, and that sphingosine kinase activity contributes to this effect. We further show that myofibroblast differentiation stimulated by OSCC cells involves the S1P2 receptor (and possibly S1PR3), although intracellular S1P targets may contribute to this effect. Whilst our study used the pharmacological modulation of S1P signalling to regulate α-SMA expression, further validation in animal models or human patient samples is needed. Nevertheless, our results highlight the S1P signalling pathway as a potential therapeutic target worthy of further investigation to target myoCAFs in OSCC.

## Supplemental Information

10.7717/peerj.21673/supp-1 Supplemental Information 1 Supplemental figures.

10.7717/peerj.21673/supp-2 Supplemental Information 2 Full blots for WBs in Figs. 1–4.

10.7717/peerj.21673/supp-3 Supplemental Information 3 MIQE checklist.

10.7717/peerj.21673/supp-4 Supplemental Information 4 Raw QPCR data.

## Additional Information and Declarations

### Data Availability

The following information was supplied regarding data availability:

The data is available in the Supplemental Files.
