# Supplementary material for: Sphingosine 1-phosphate signalling contributes to transforming growth factor-β1-induced myofibroblast differentiation in oral cancer
Source: PeerJ. 2026 Sep 7;14:e21673. doi: 10.7717/peerj.21673 (PMC13564403; doi:10.7717/peerj.21673)
Supplement: Supplementary file 1 [file peerj-14-21673-s001.pdf]

Supplementary Figures

A

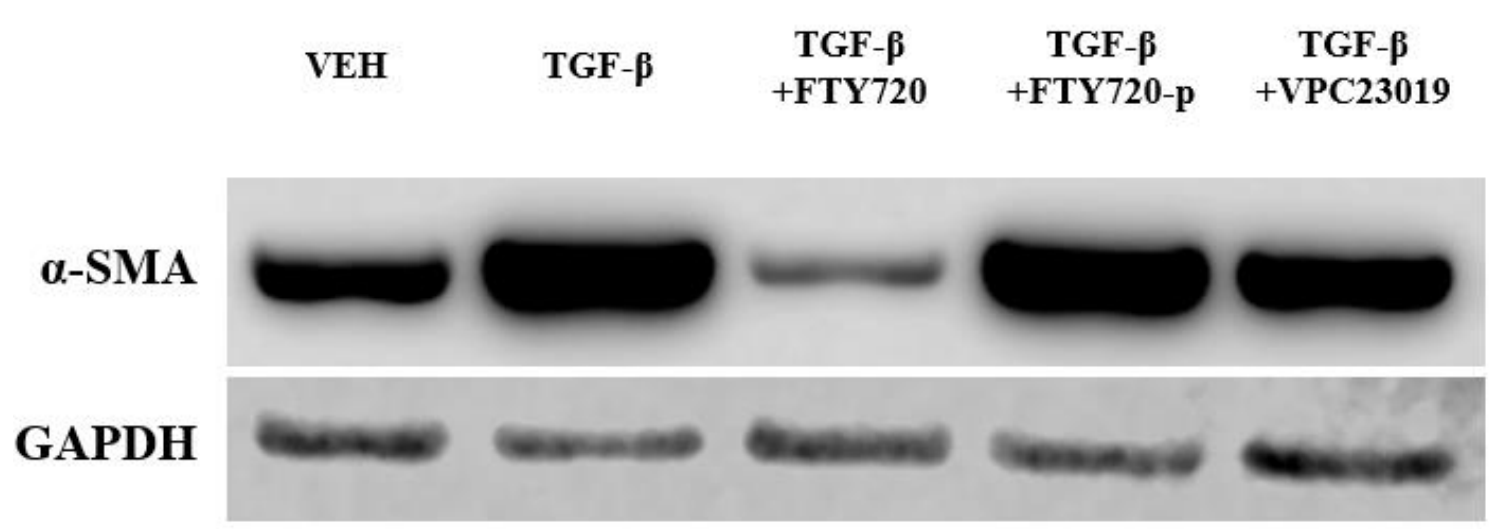

Figure S1: Effect of inhibiting S1PRs in NHOF1. (A) Treatment with FTY720-p, inhibitor of S1PR1, 2, 4, 5, and VPC23019, inhibitor of S1P1/S1PR3, did not decrease the α-SMA protein levels in NHOFs induced by TGF-β.

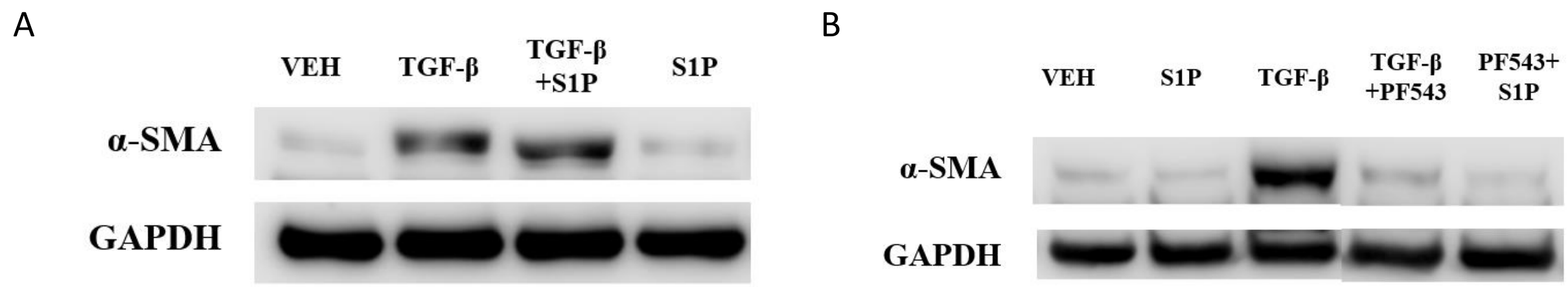

Figure S2: Effects of exogenous S1P on NHOF1. (A) Western blotting images showed S1P failed to exacerbate α-SMA protein expression induced by TGF-β in NHOF. (B) Inhibition of SK1 using PF543 reduced α-SMA protein levels in NHOF and this effect cannot be rescued by exogenous S1P treatment.

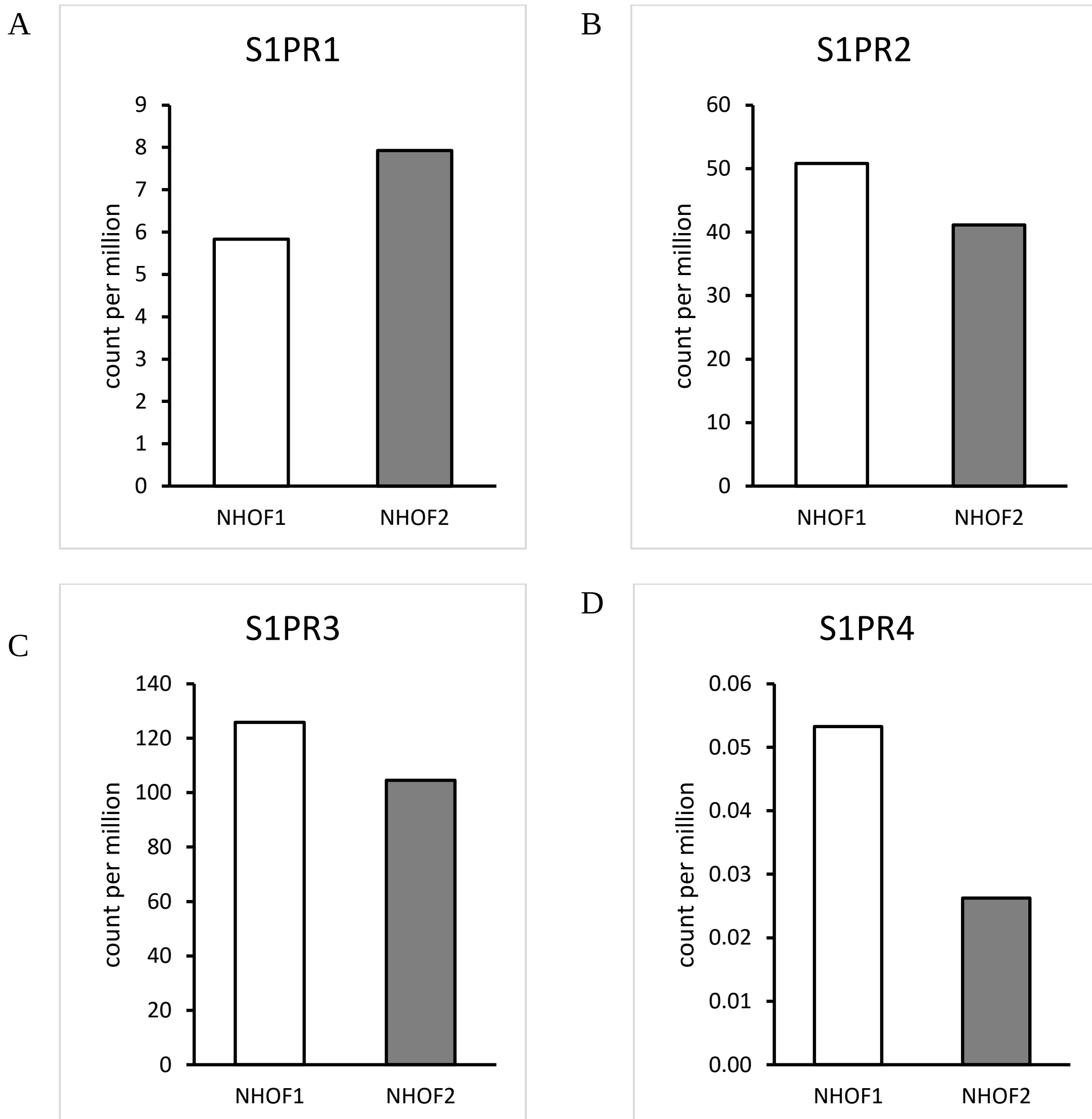

A
S1PR1
count per million
0
1
2
3
4
5
6
7
8
9
NHOF1
NHOF2
B
S1PR2
count per million
0
10
20
30
40
50
60
NHOF1
NHOF2
C
S1PR3
count per million
0
20
40
60
80
100
120
140
NHOF1
NHOF2
D
S1PR4
count per million
0.00
0.01
0.02
0.03
0.04
0.05
0.06
NHOF1
NHOF2

E

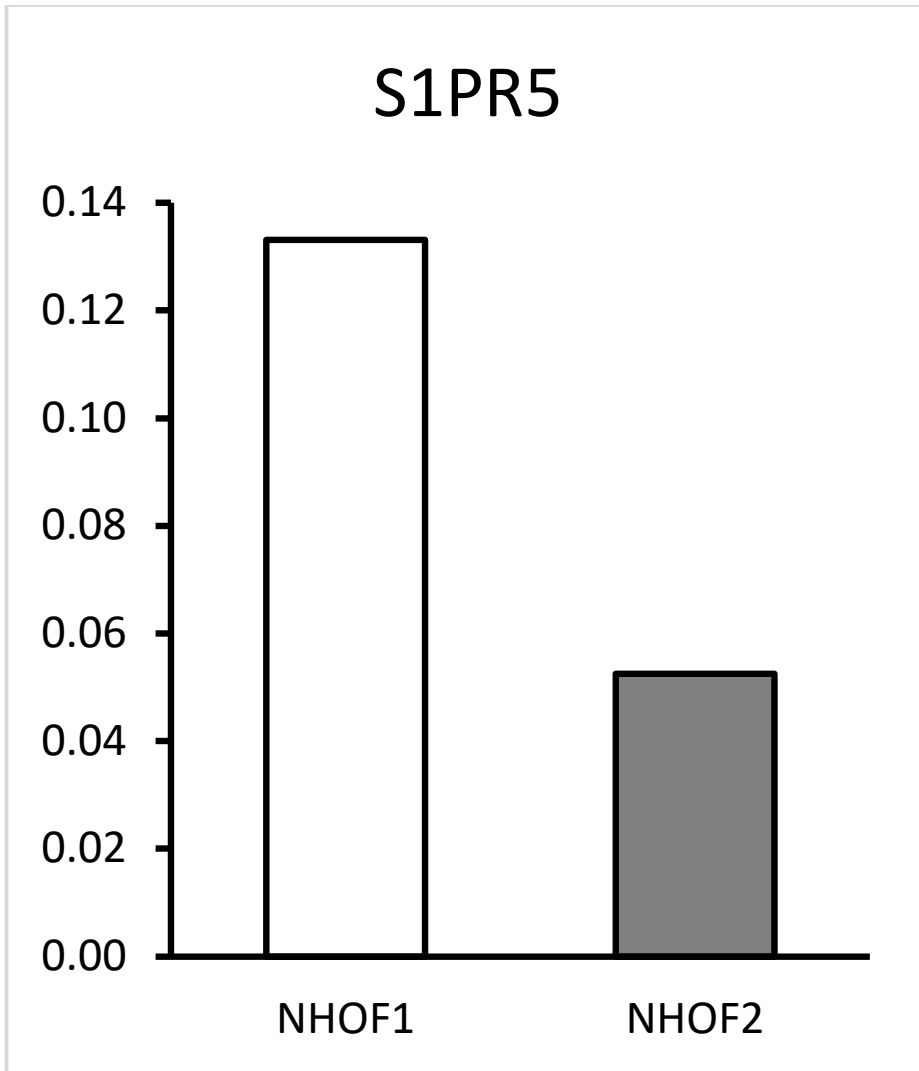

Figure S3: RNAseq analysis showing expression of sphingosine receptor subtypes in NHOFs

A. NHOF1

- JTE013	+ JTE013
1	0.545232
1	0.140678
1	0.520384

- JTE013	+ JTE013
1	0.391325
	0.407211
1	0.272571

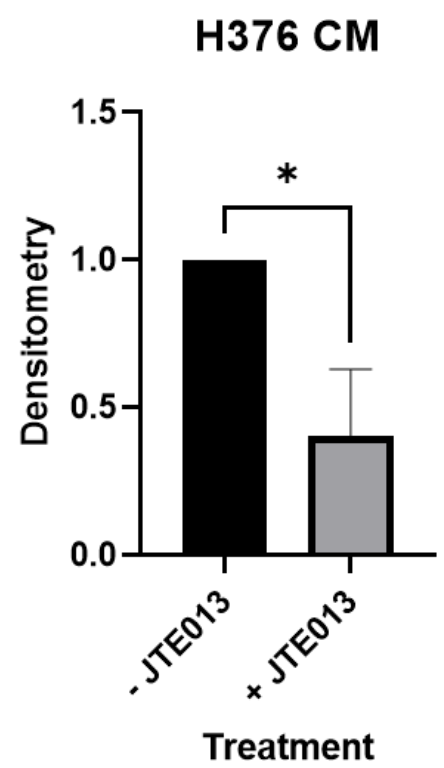

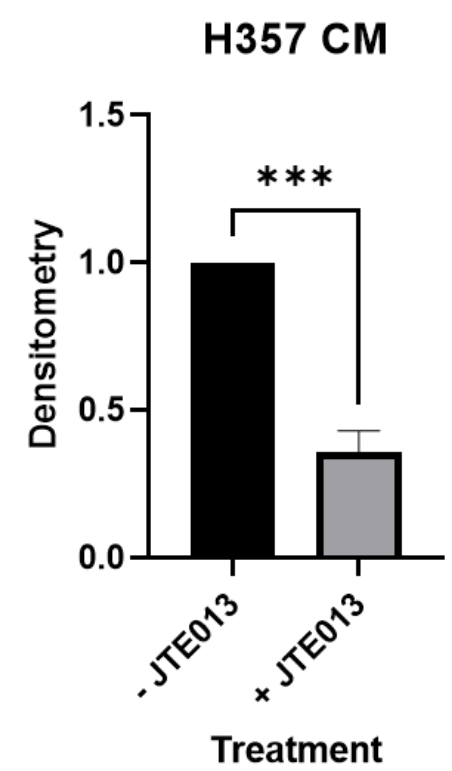

B. NHOF2

- JTE013	+ JTE013
1	0.507289
1	0.337082
1	0.331914

- JTE013	+ JTE013
1	0.465552
1	0.849678
1	0.487613

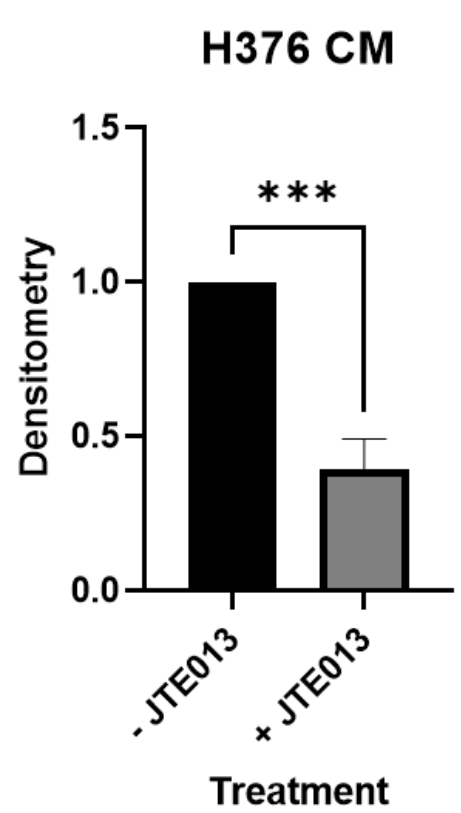

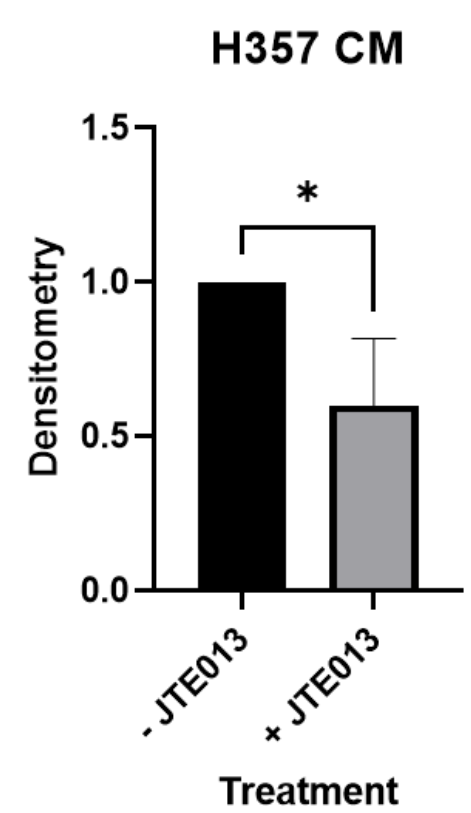

Figure S4: Effect of JTE013 on α-SMA levels induced by OSCC CM in NHOF1 (A) and NHOF2 (B). Combined densitometry data from 3 independent experiments are shown. Normalised values are shown in the tables. Student's t-test *p<0.05; ***p<0.001.

A. **NHOF1**

-	CYM5520	CYM5541
1	7.171051	4.225394
1	1.619995	0.971519
1	2.98101	1.822967

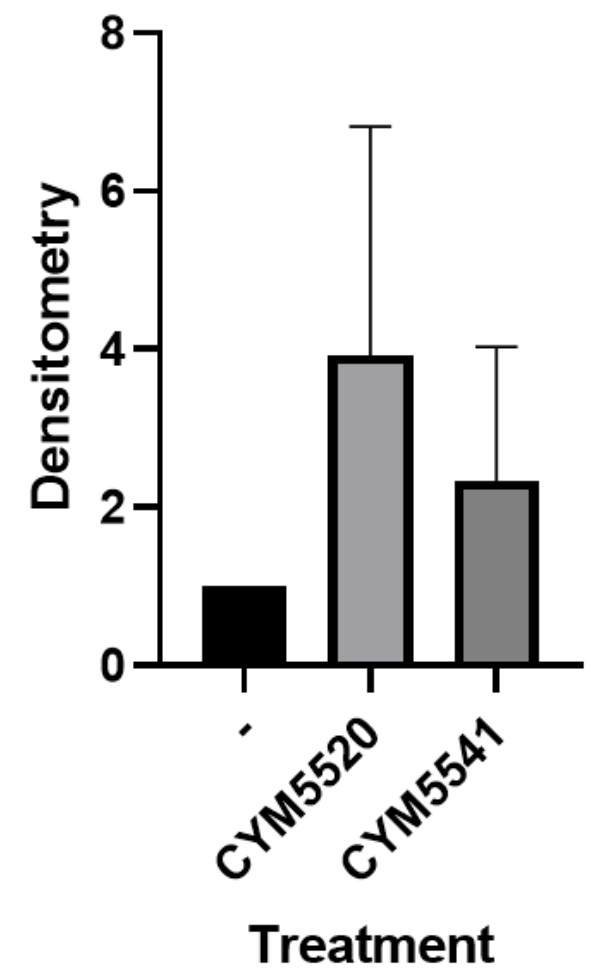

B. **NHOF2**

-	CYM5520	CYM5541
1	6.502695	5.772949
1	2.624449	1.807279
1	1.344805	0.599707

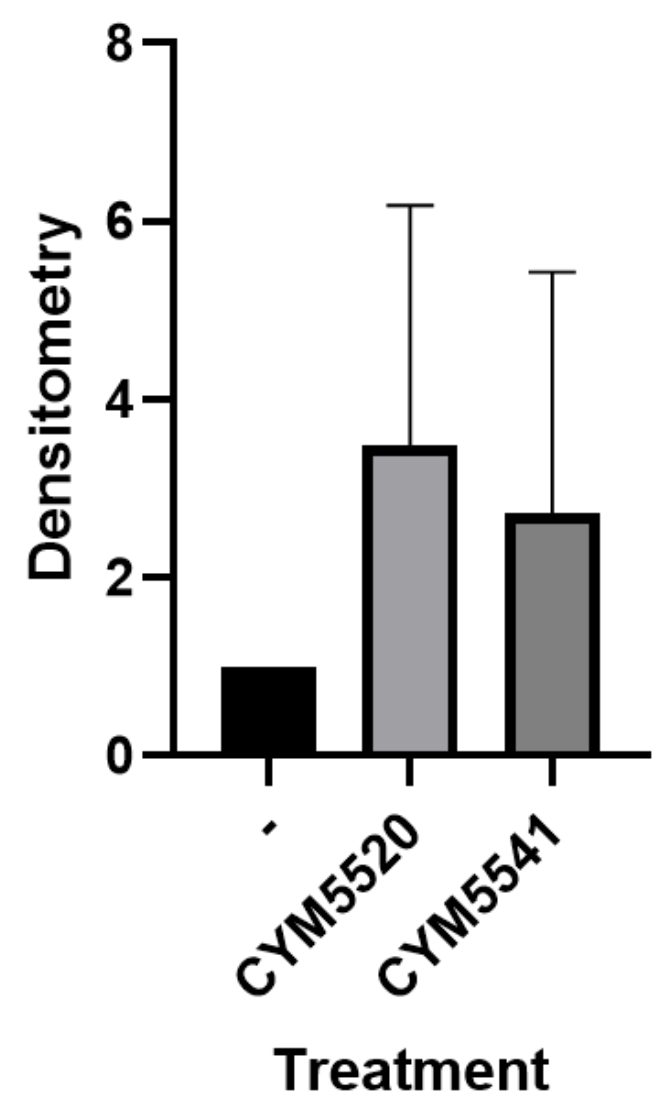

Figure S5: Effect of CYM5520 (S1PR2 agonist) and CYM5541 (S1P3 agonist) on α-SMA levels in NHOF1 (A) and NHOF2 (B). Combined densitometry data from 3 independent experiments are shown. Normalised values are shown in the tables. α-SMA levels were increased by both compounds, but the effect of CYM 5520 was more consistent. Student's t-test, non-significant.
