# Supplementary material for: Sphingosine 1-phosphate signalling contributes to transforming growth factor-β1-induced myofibroblast differentiation in oral cancer
Source: PeerJ. 2026 Sep 7;14:e21673. doi: 10.7717/peerj.21673 (PMC13564403; doi:10.7717/peerj.21673)

Precision Plus Protein™ All Blue Prestained Protein Standards #1610373

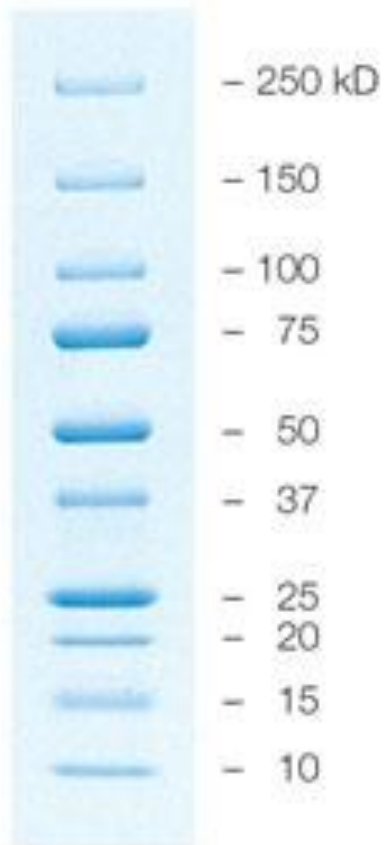

PM1700 SMOBIO

ExcelBandTM All Blue Broad Range Protein Marker

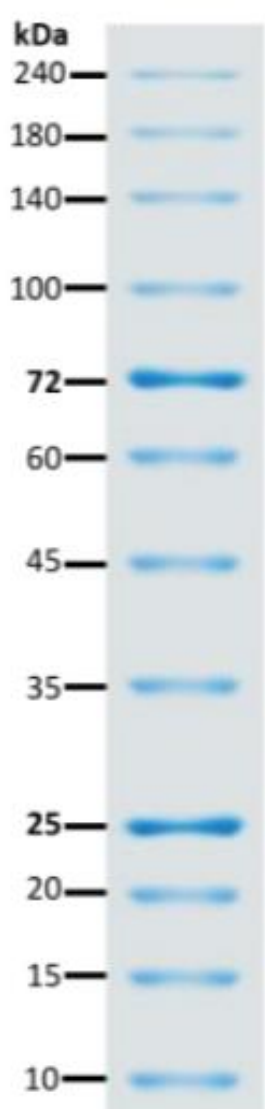

Uncropped blots of Figure 1A

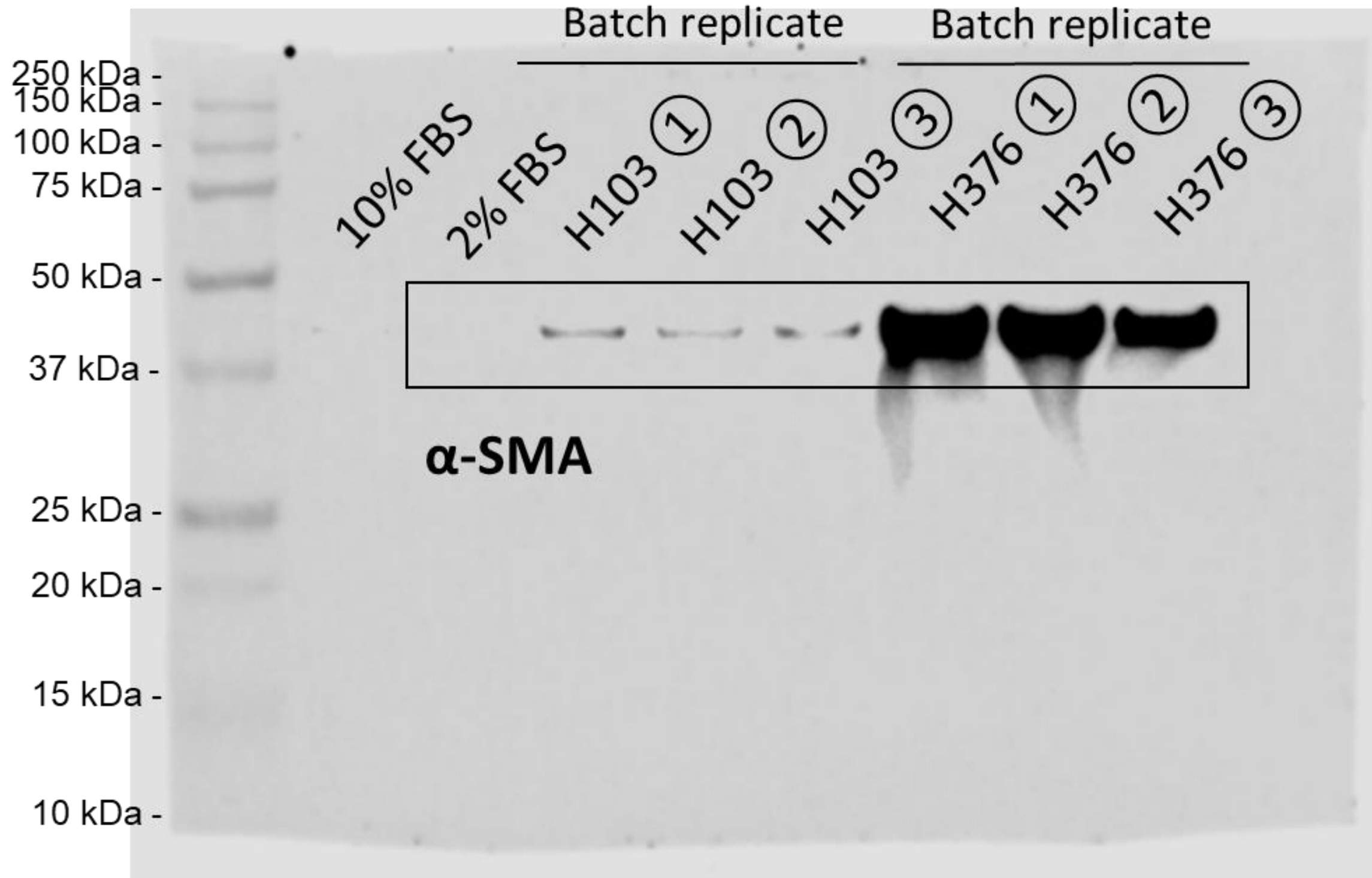

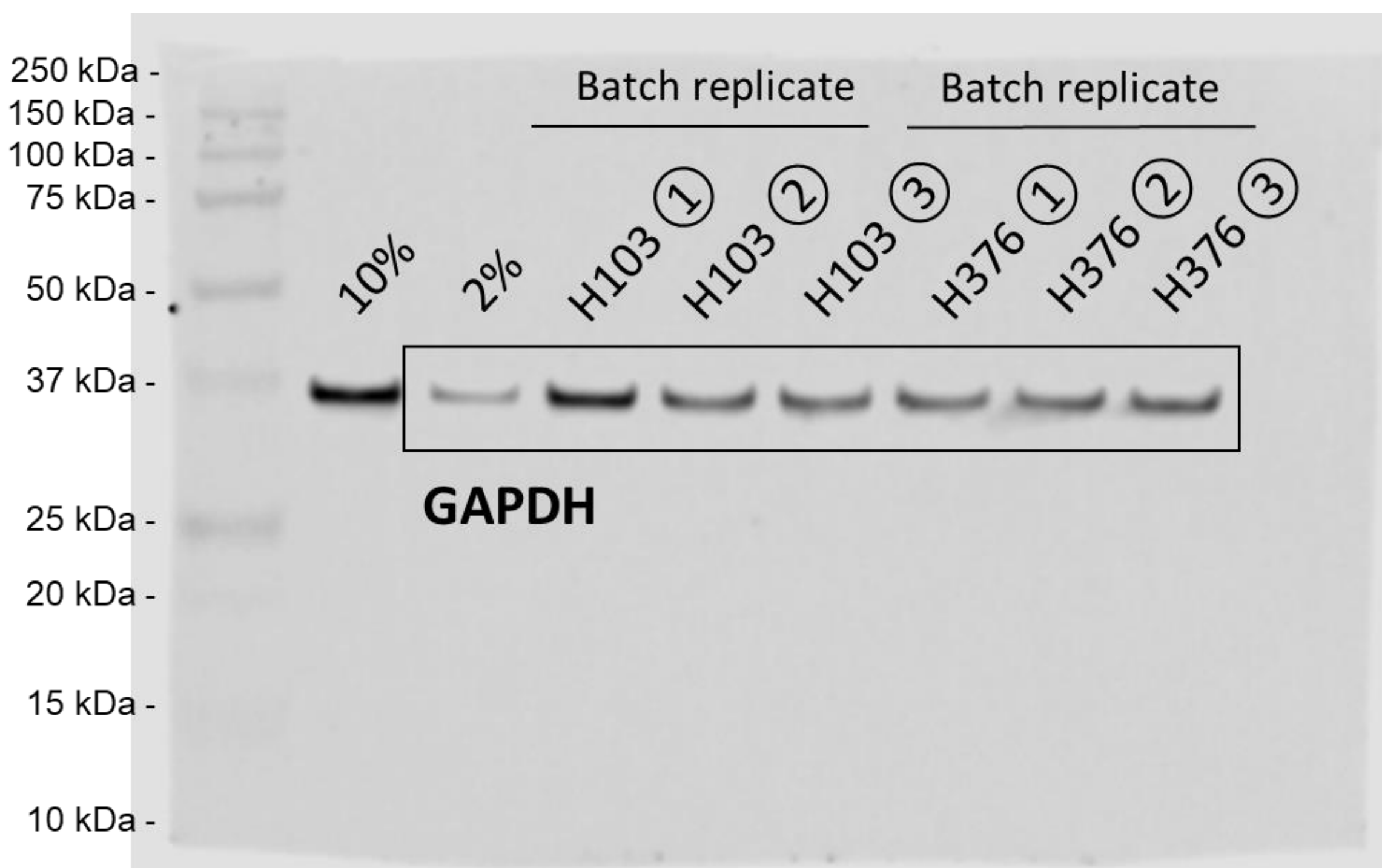

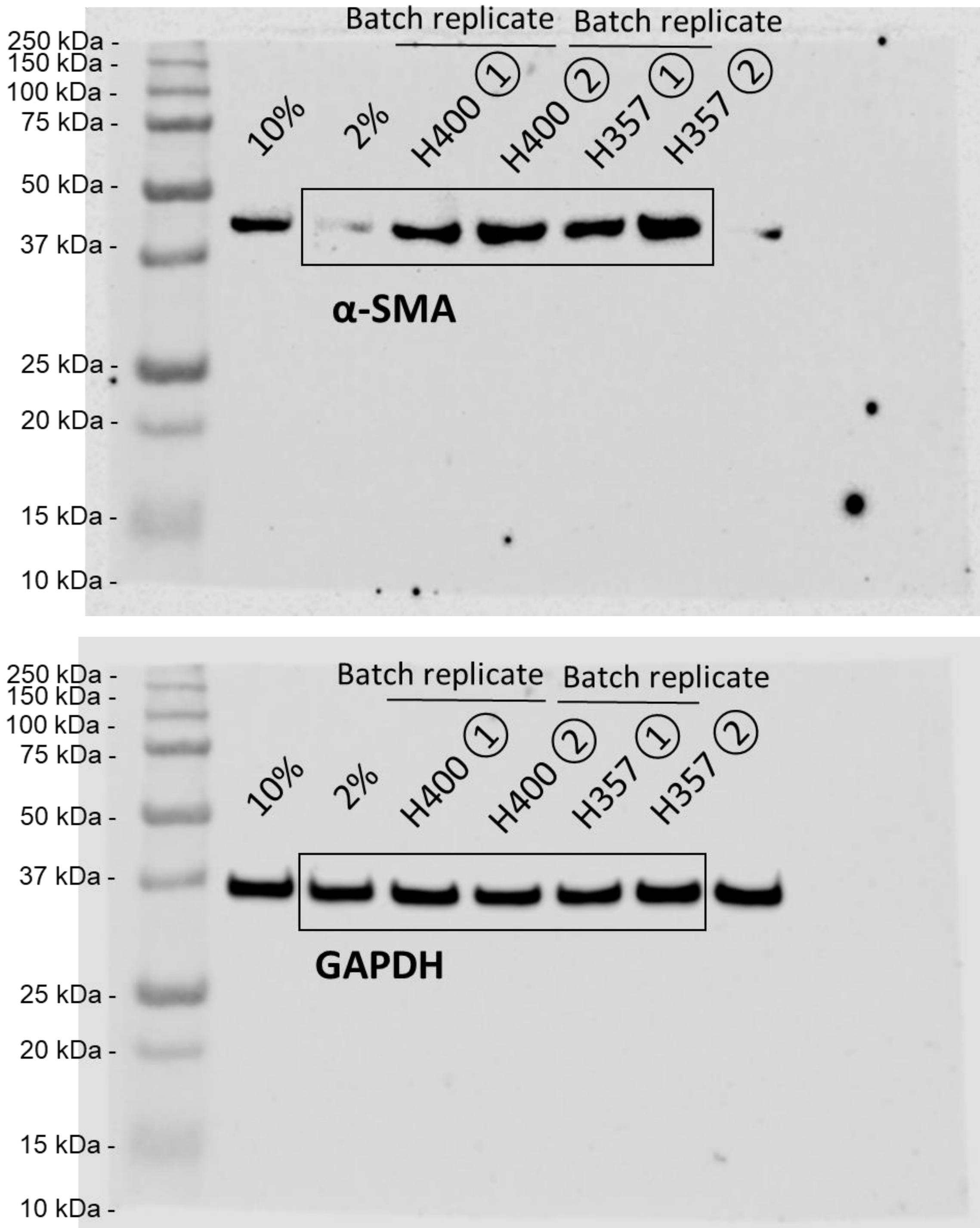

250 kDa -
150 kDa -
100 kDa -
75 kDa -
50 kDa -
37 kDa -
25 kDa -
20 kDa -
15 kDa -
10 kDa -
Batch replicate
Batch replicate
10%
2%
H400 1
H400 2
H357 1
H357 2
α-SMA
250 kDa -
150 kDa -
100 kDa -
75 kDa -
50 kDa -
37 kDa -
25 kDa -
20 kDa -
15 kDa -
10 kDa -
Batch replicate
Batch replicate
10%
2%
H400 1
H400 2
H357 1
H357 2
GAPDH

Uncropped blots of Figure 1B

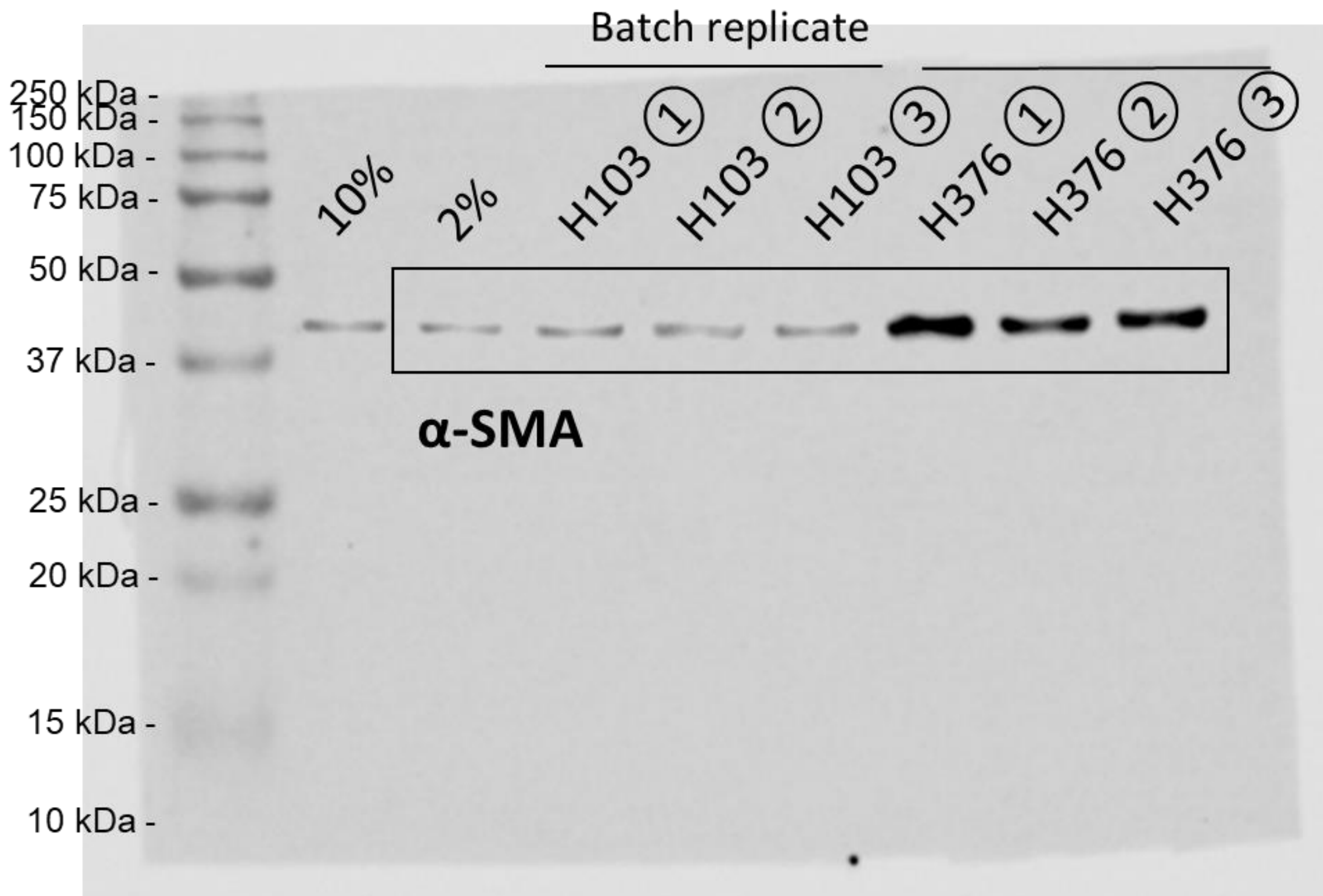

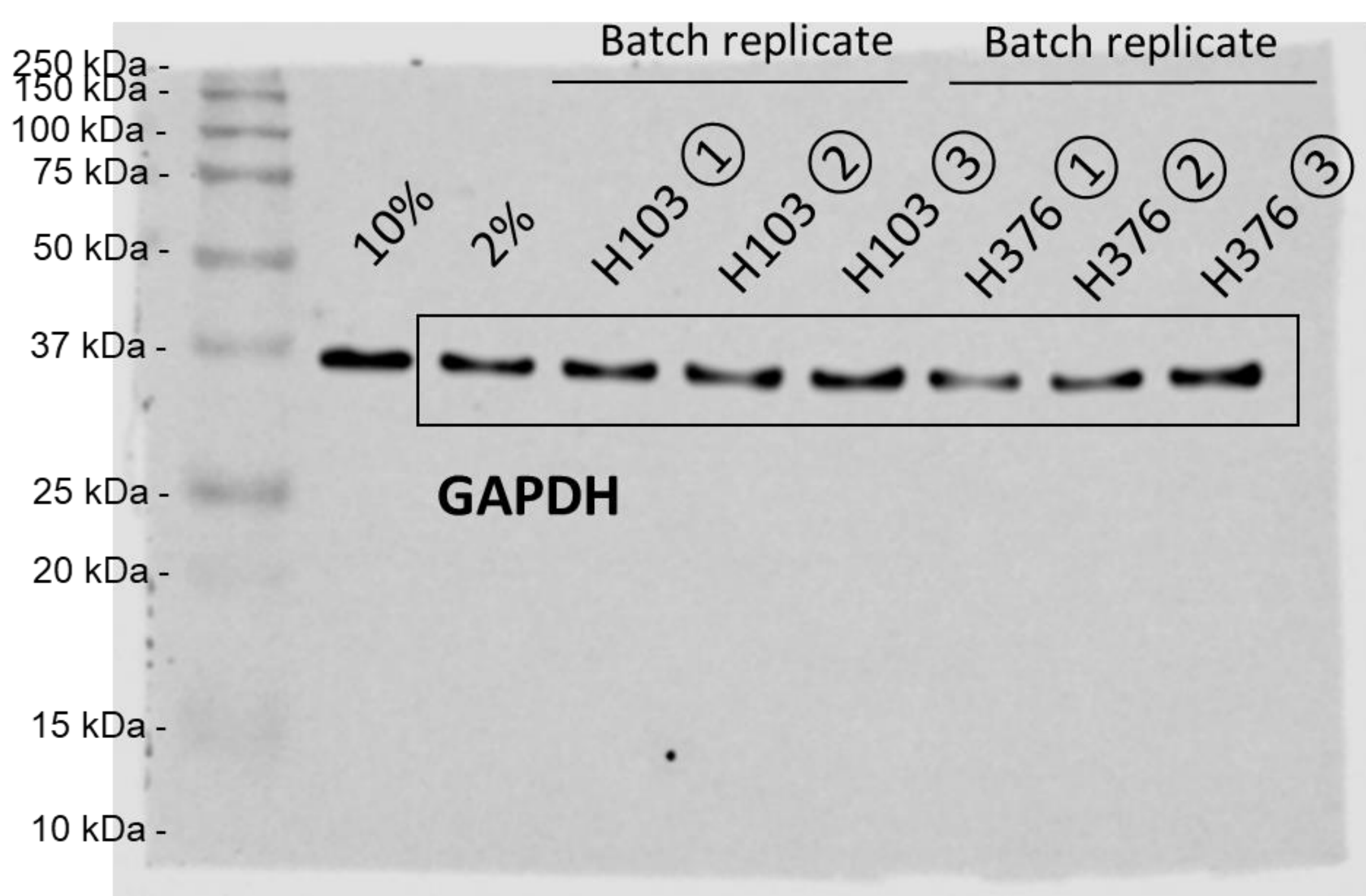

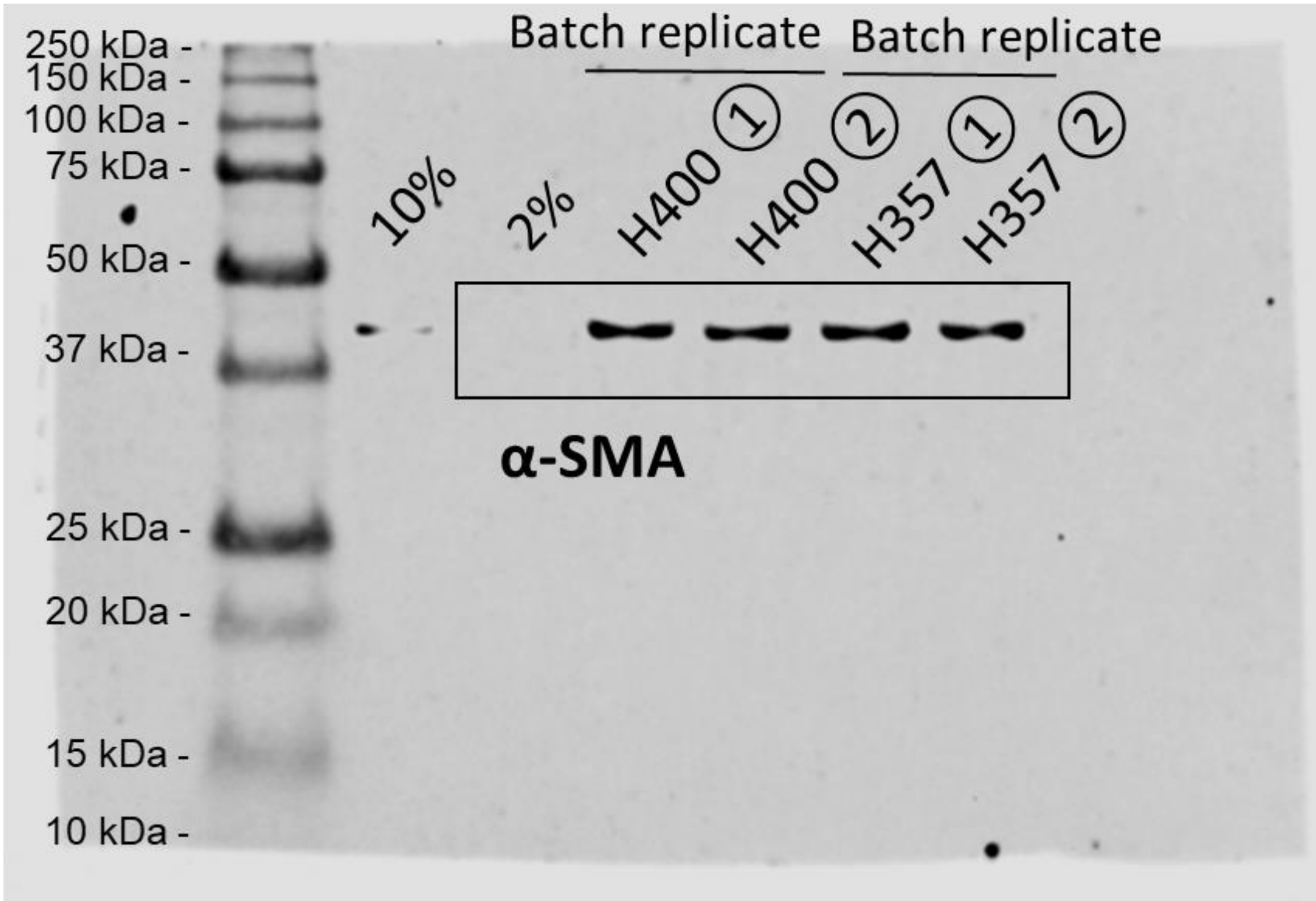
250 kDa -
150 kDa -
100 kDa -
75 kDa -
50 kDa -
37 kDa -
25 kDa -
20 kDa -
15 kDa -
10 kDa -
Batch replicate
Batch replicate
10%
2%
H400 ①
H400 ②
H357 ①
H357 ②
α-SMA

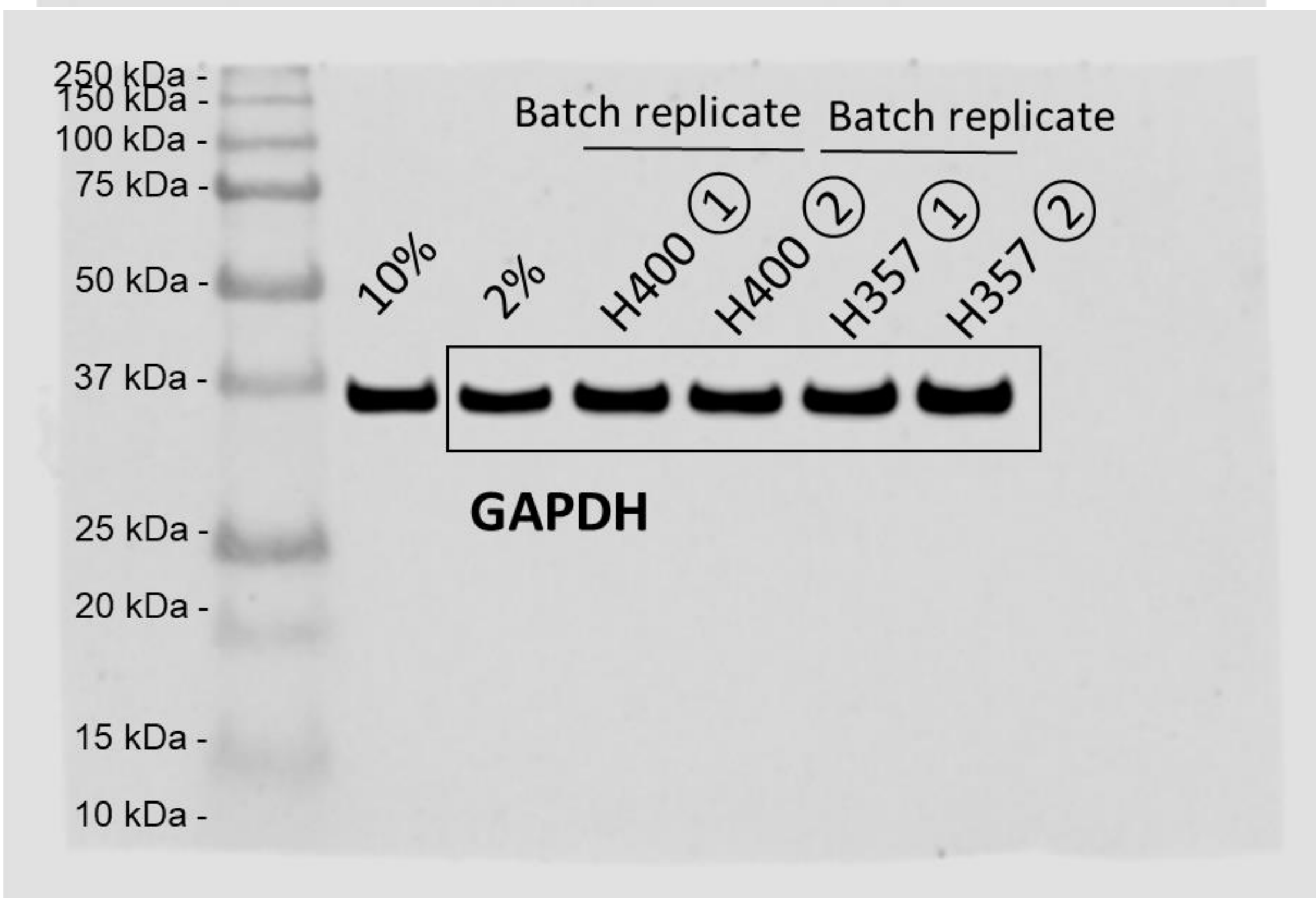
250 kDa -
150 kDa -
100 kDa -
75 kDa -
50 kDa -
37 kDa -
25 kDa -
20 kDa -
15 kDa -
10 kDa -
Batch replicate
Batch replicate
10%
2%
H400 ①
H400 ②
H357 ①
H357 ②
GAPDH

Uncropped blots of Figure 1C

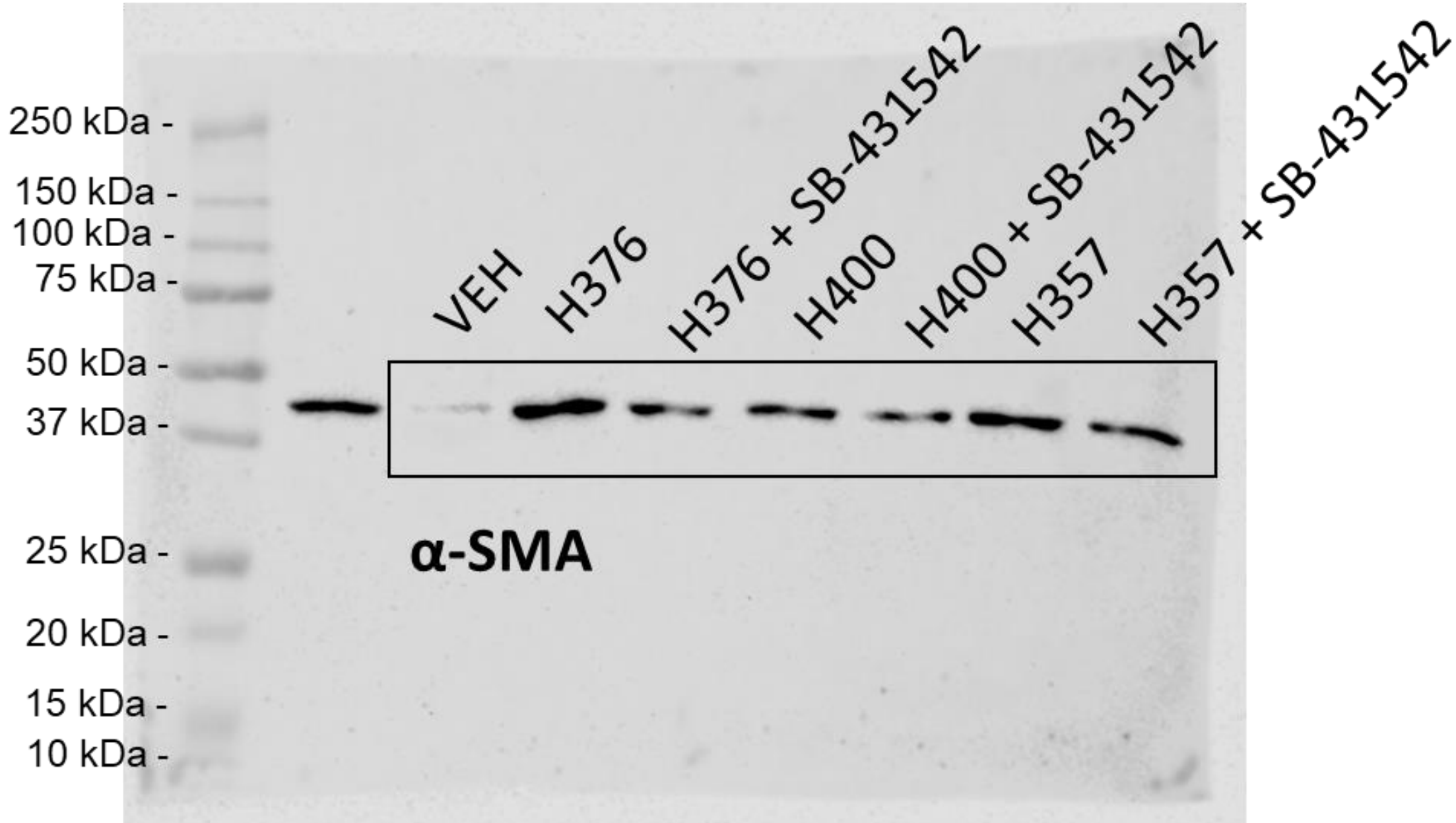

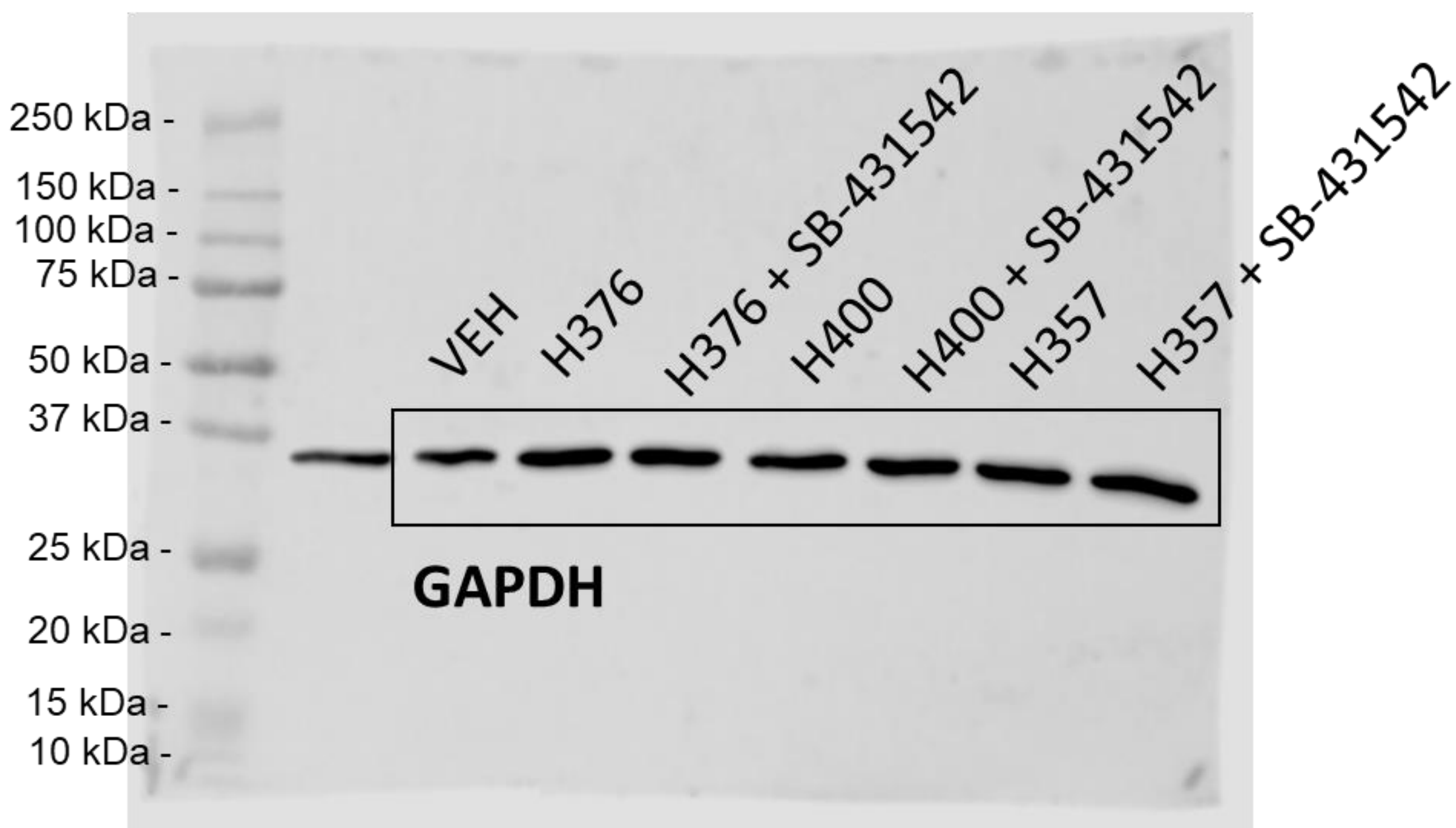

Uncropped blots of Figure 1D

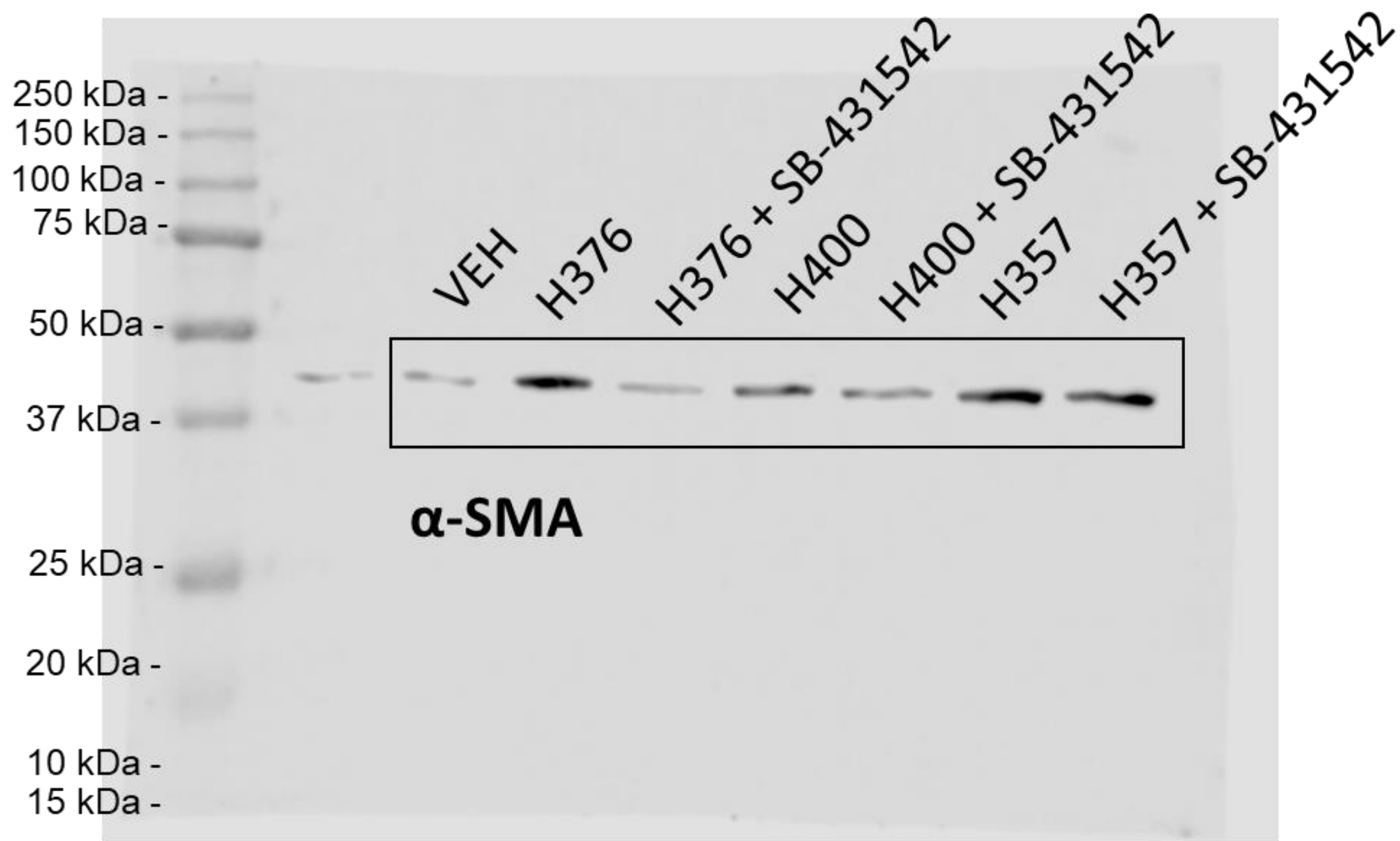

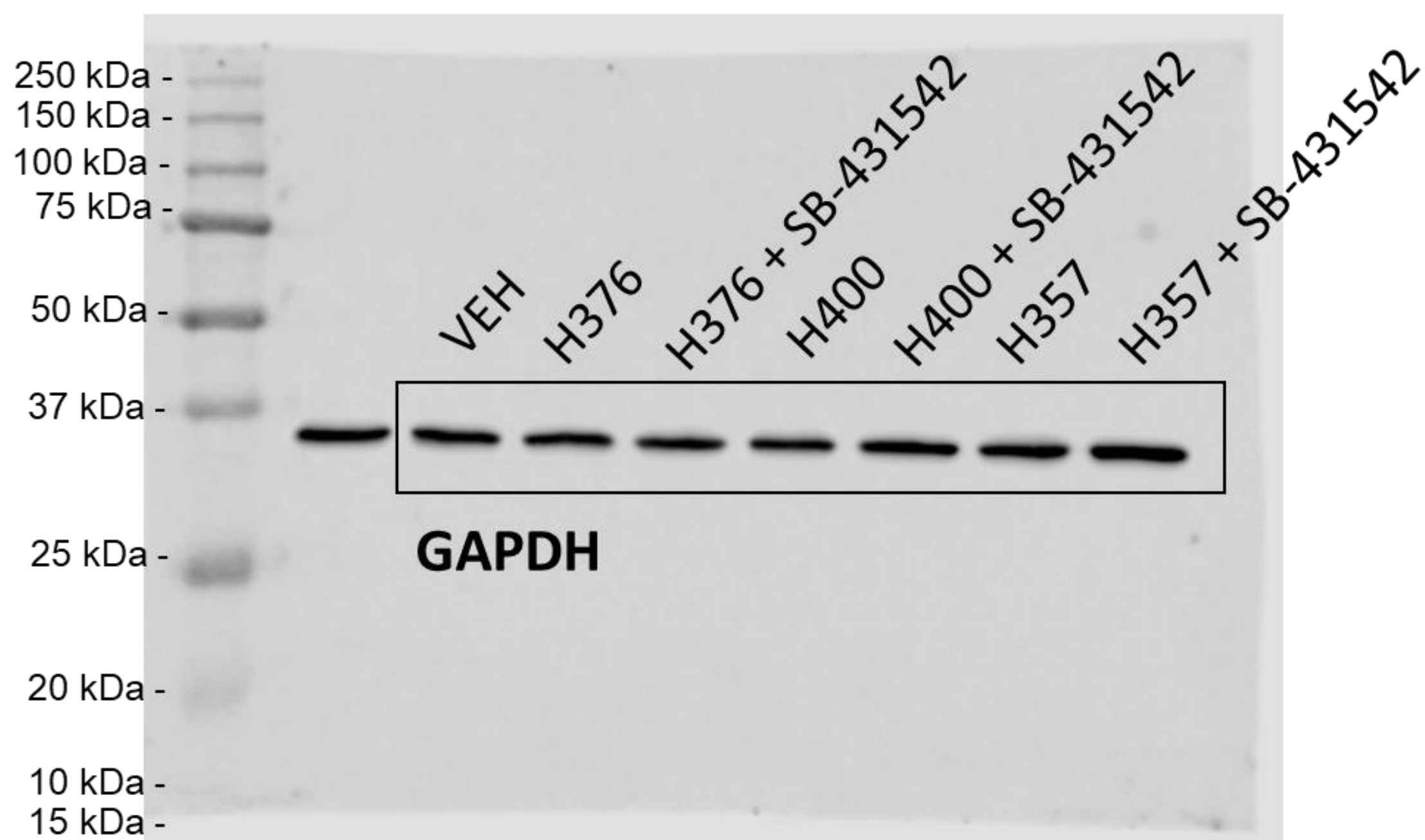

Uncropped blots of Figure 2D

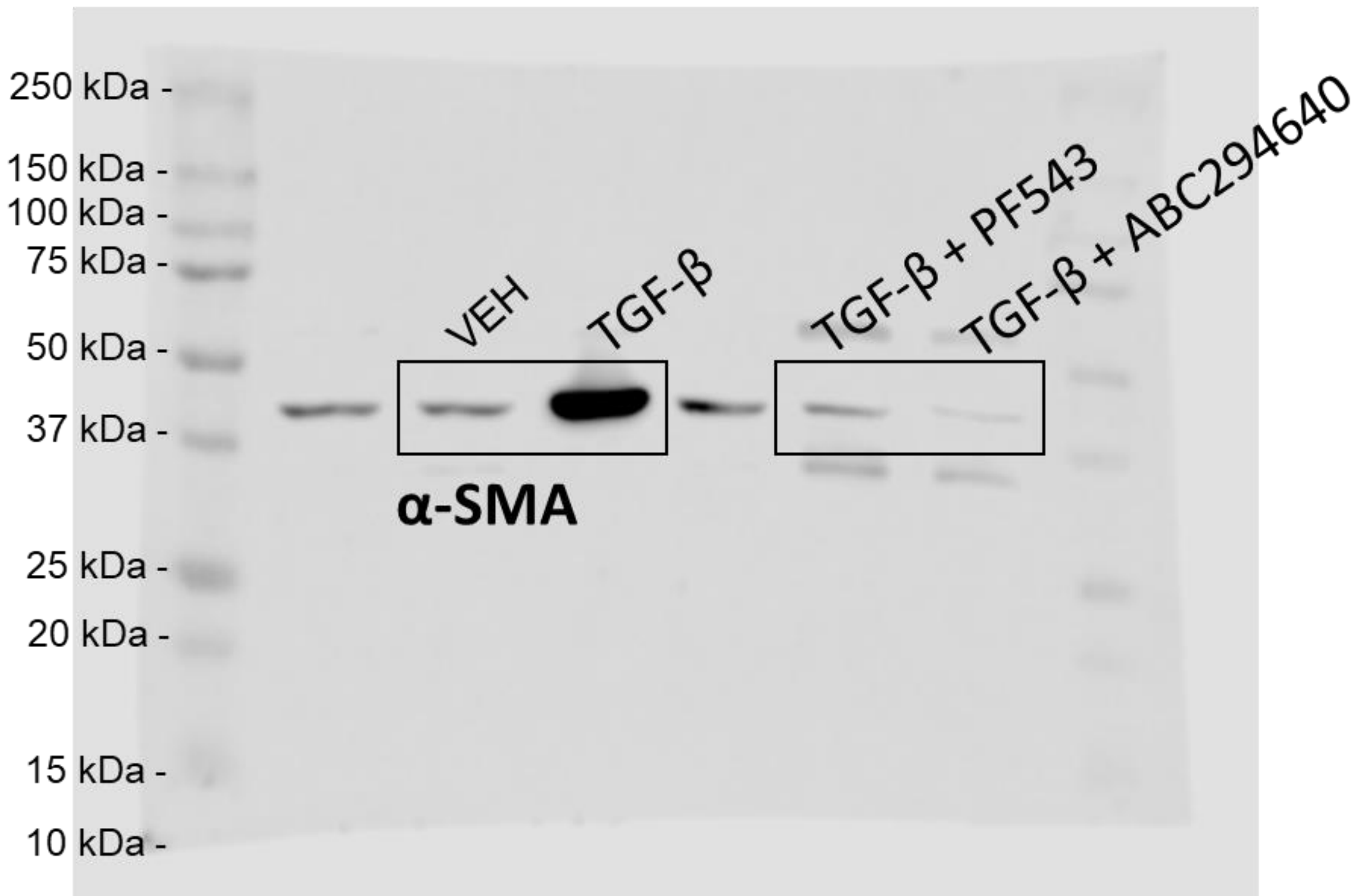

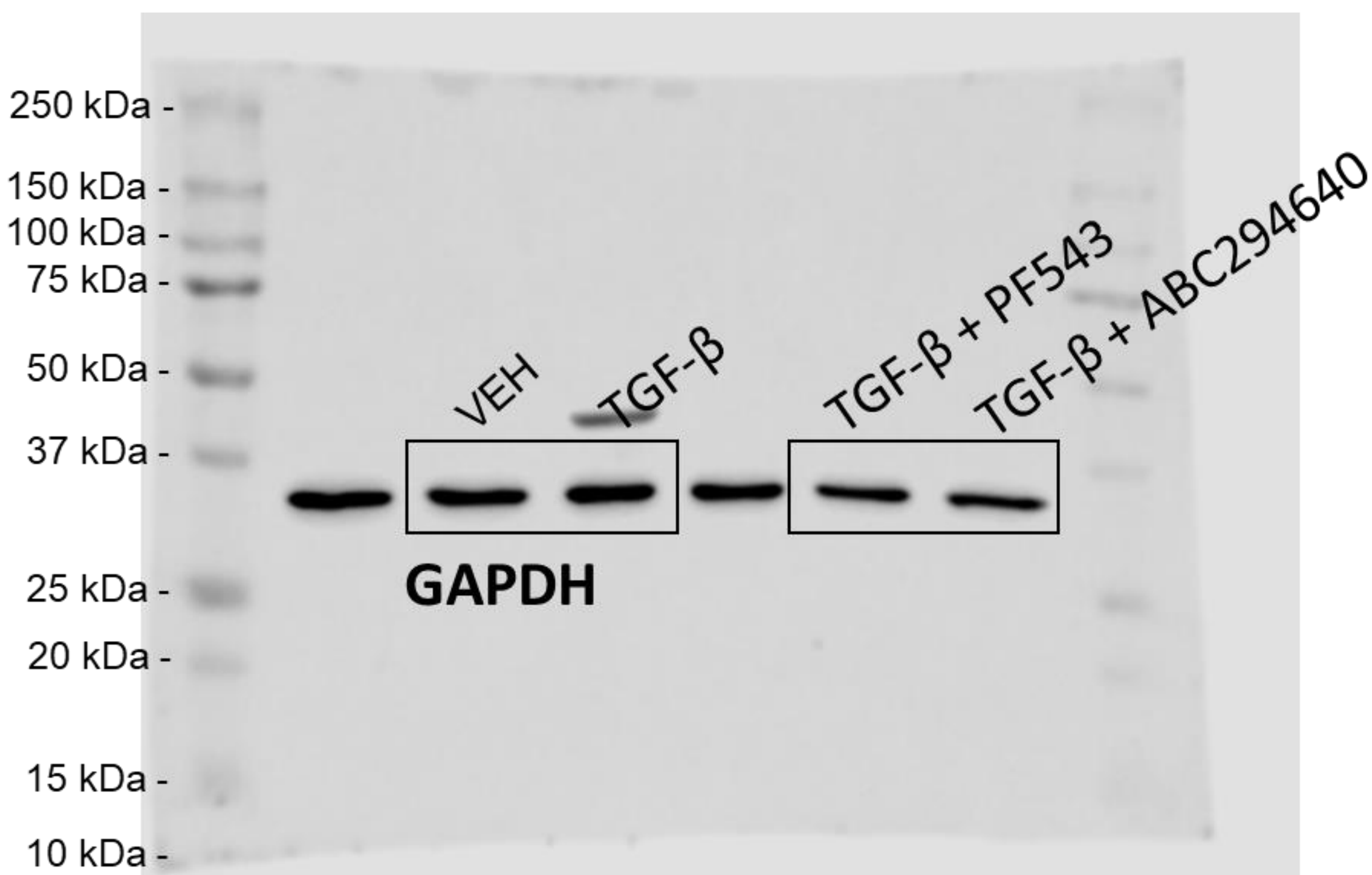

Uncropped blots of Figure 2E

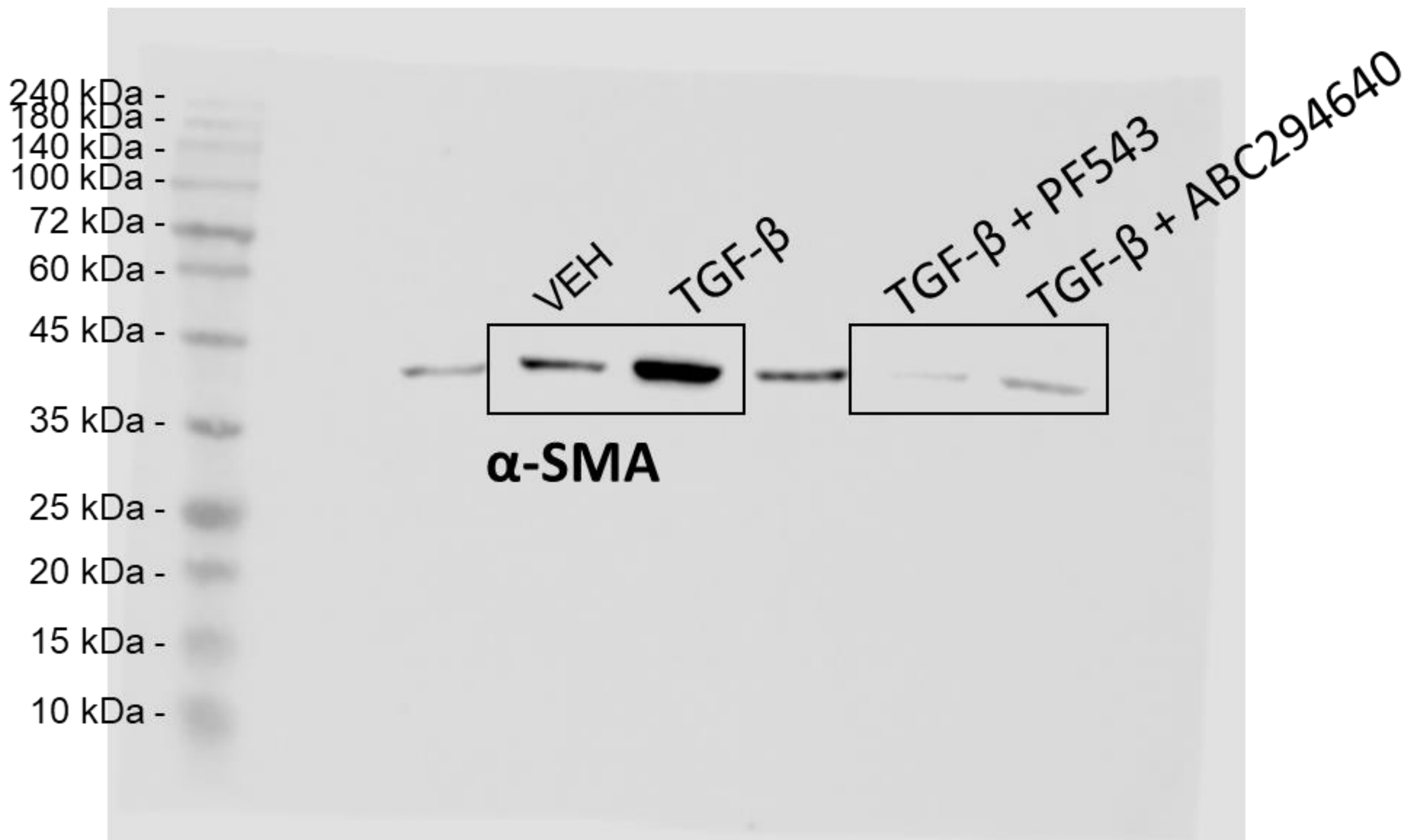

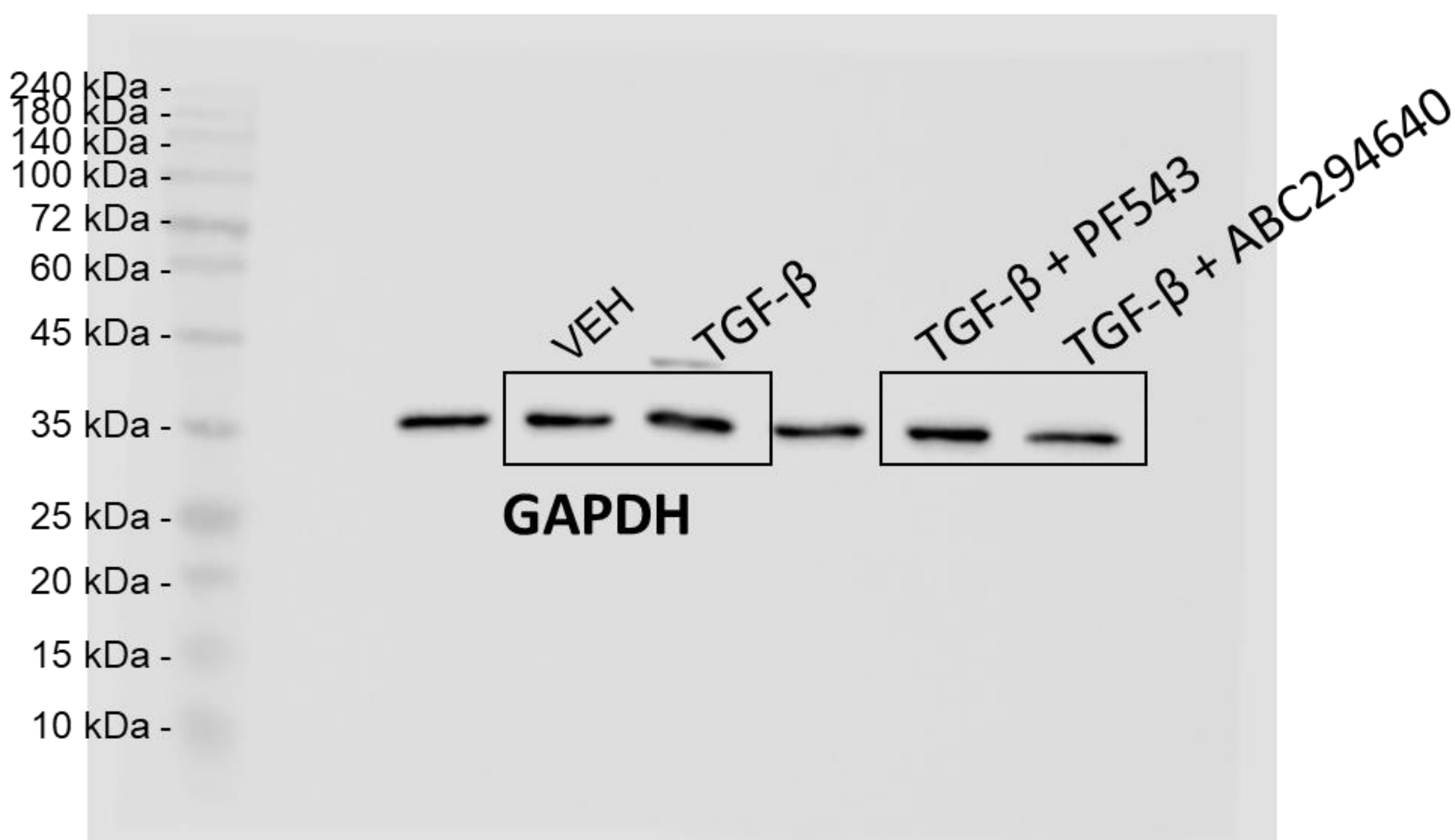

Uncropped blots of Figure 2F

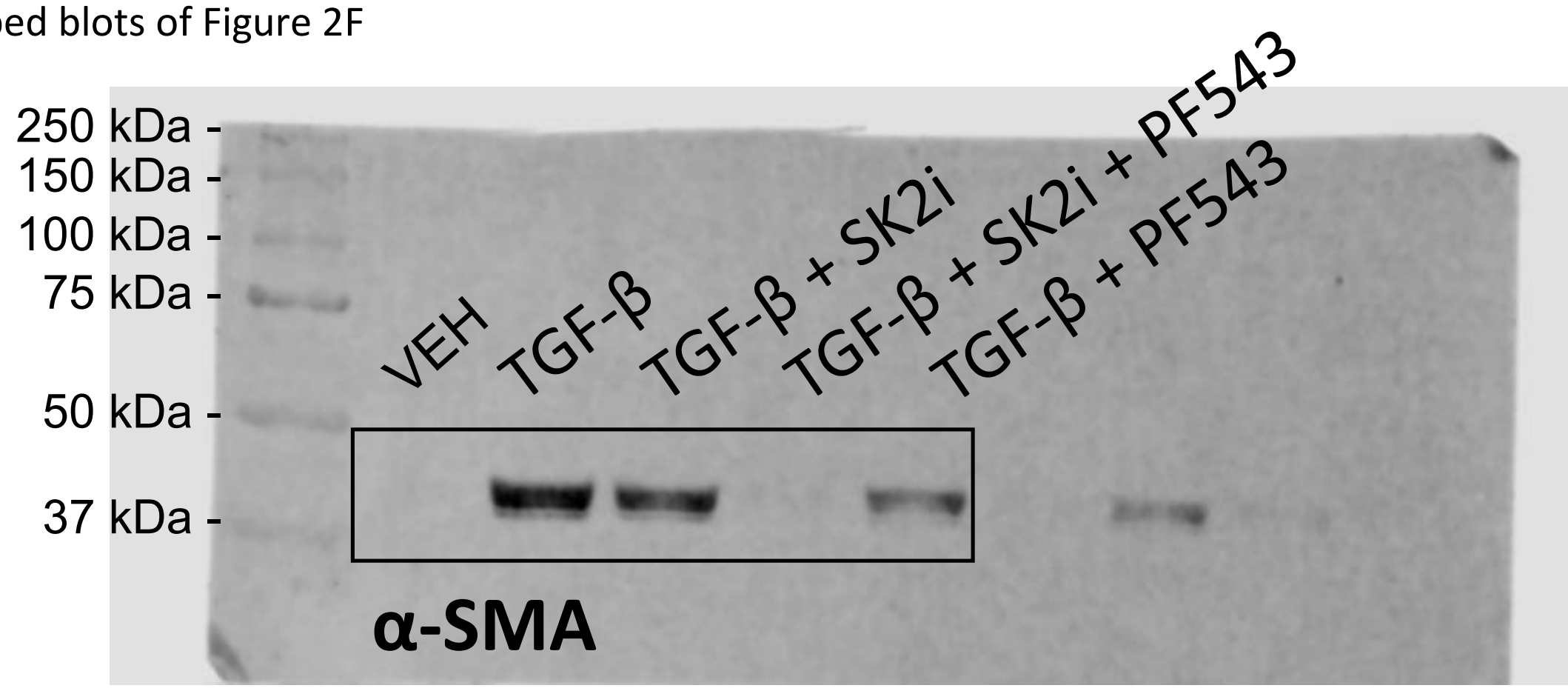

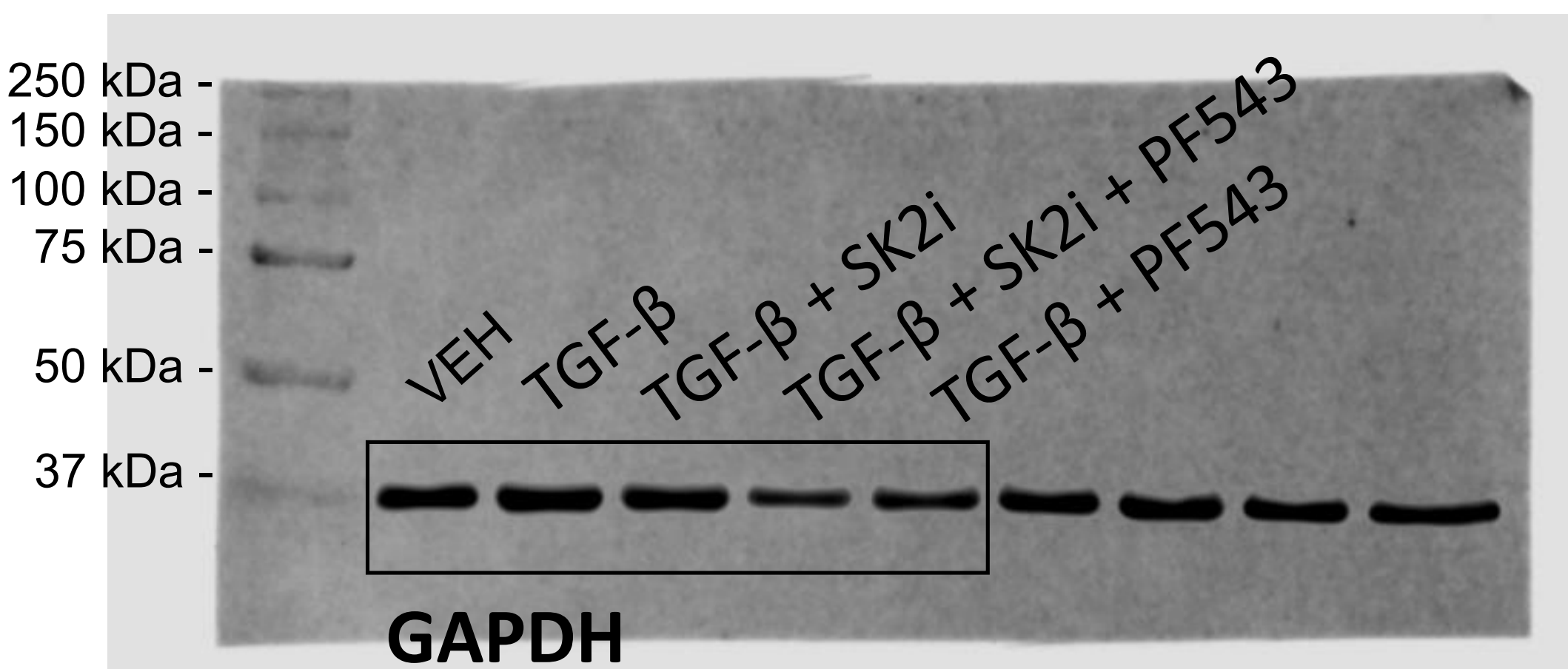

Uncropped blots of Figure 2G

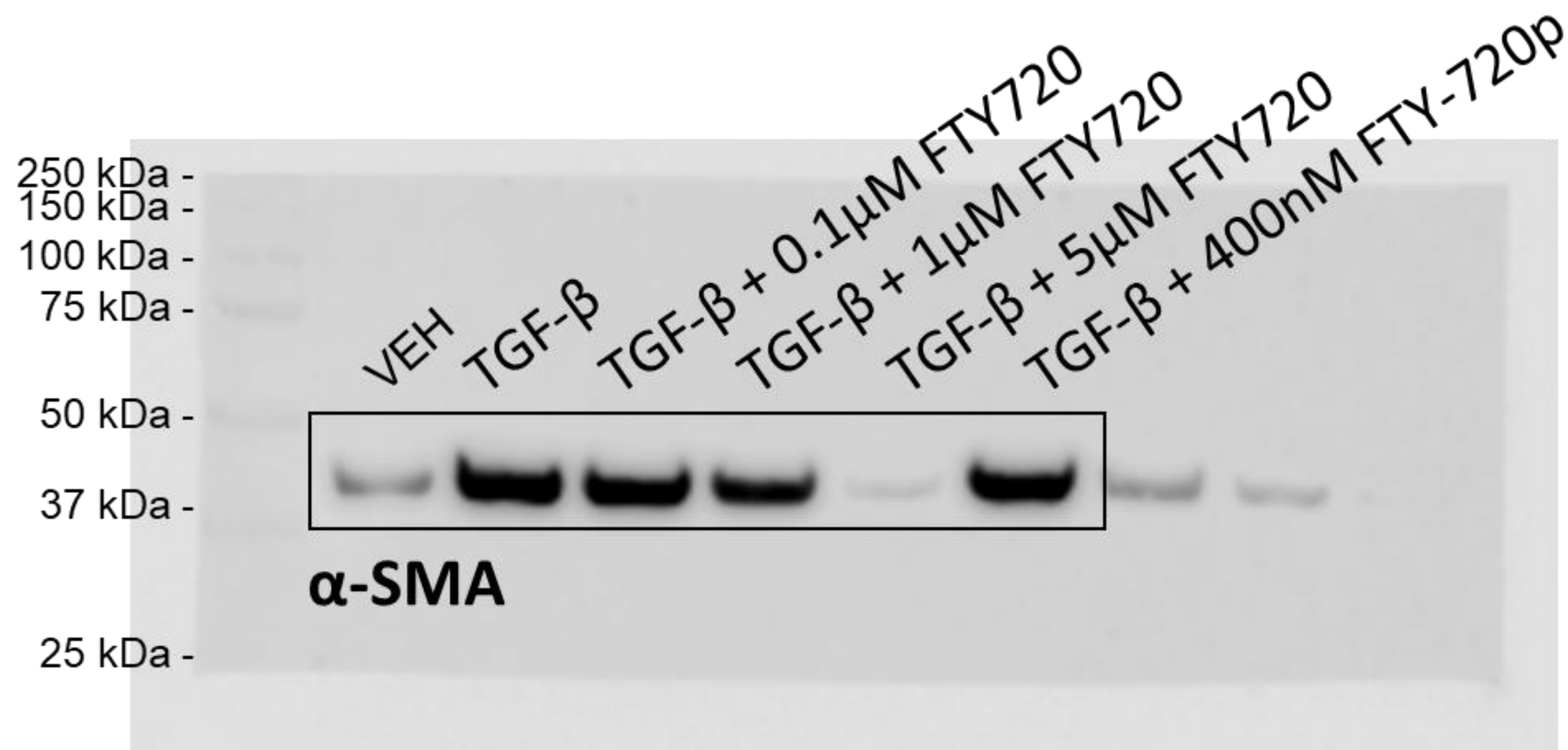

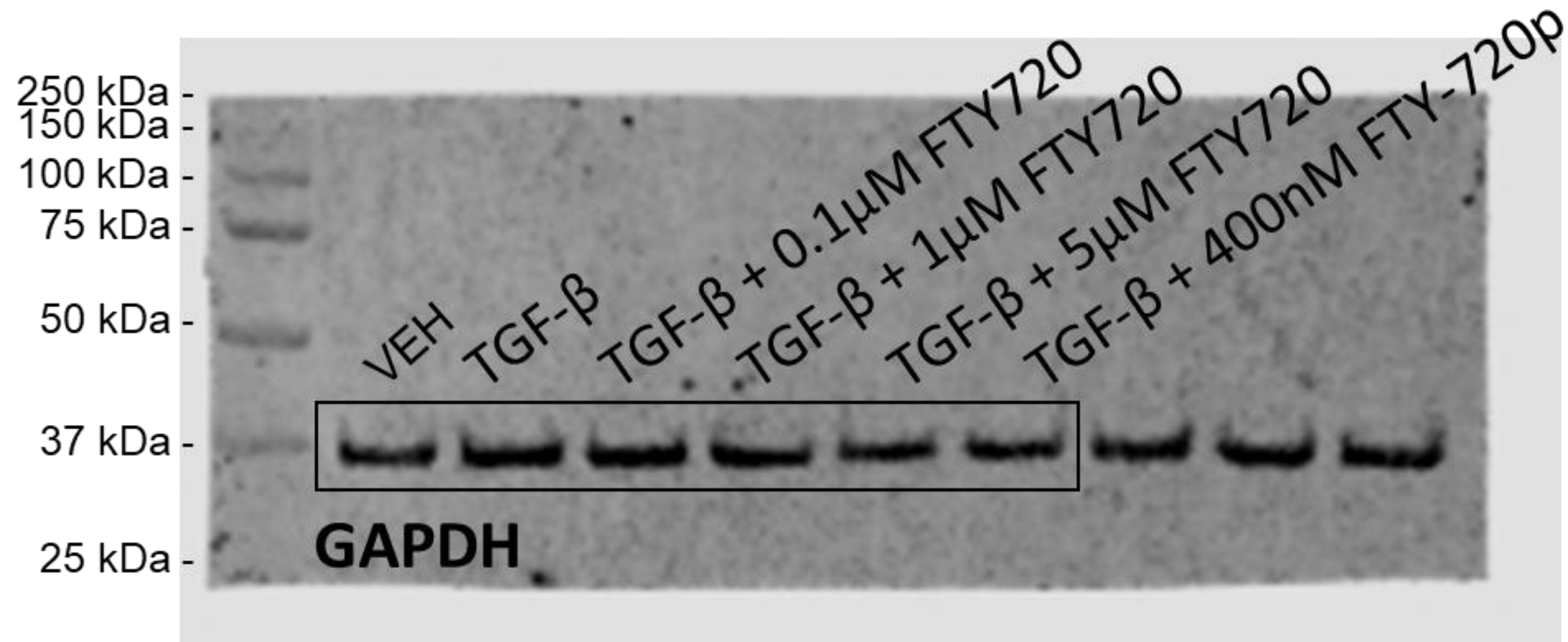

Uncropped blots of Figure 2H

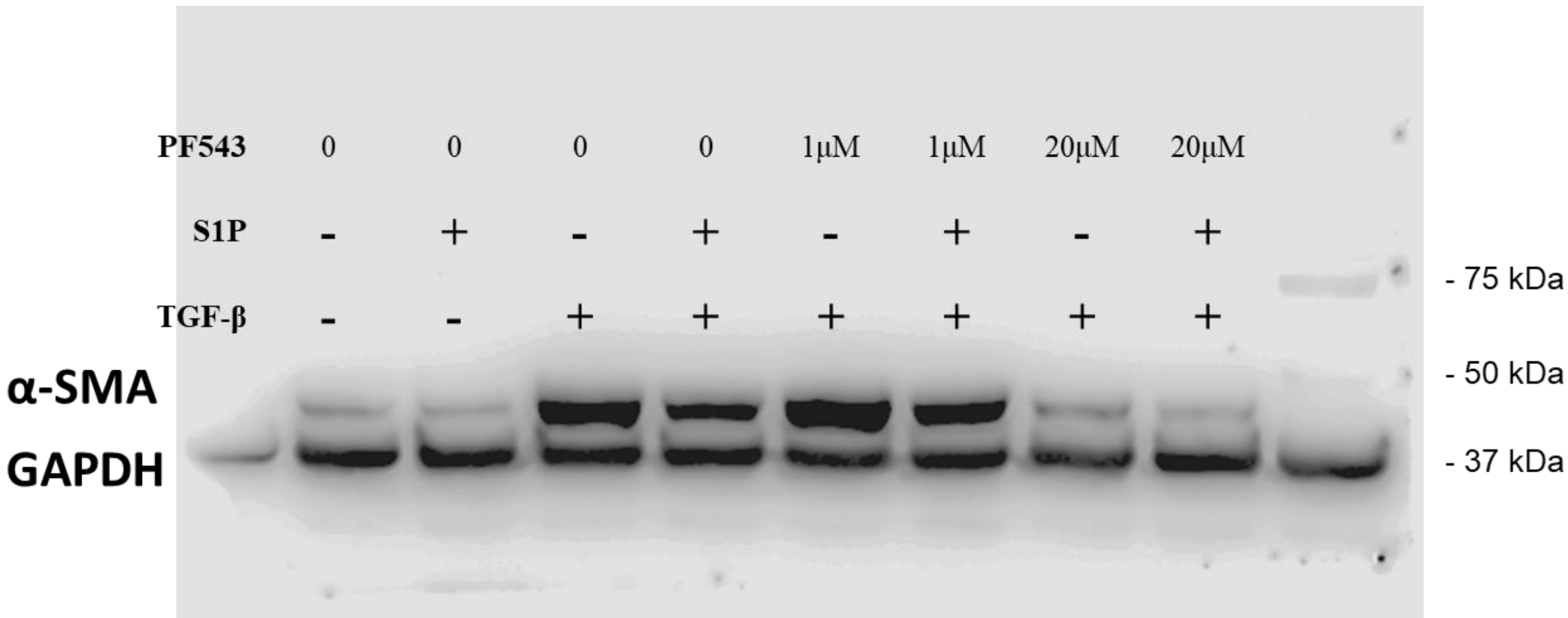

Uncropped blots of Figure 3A

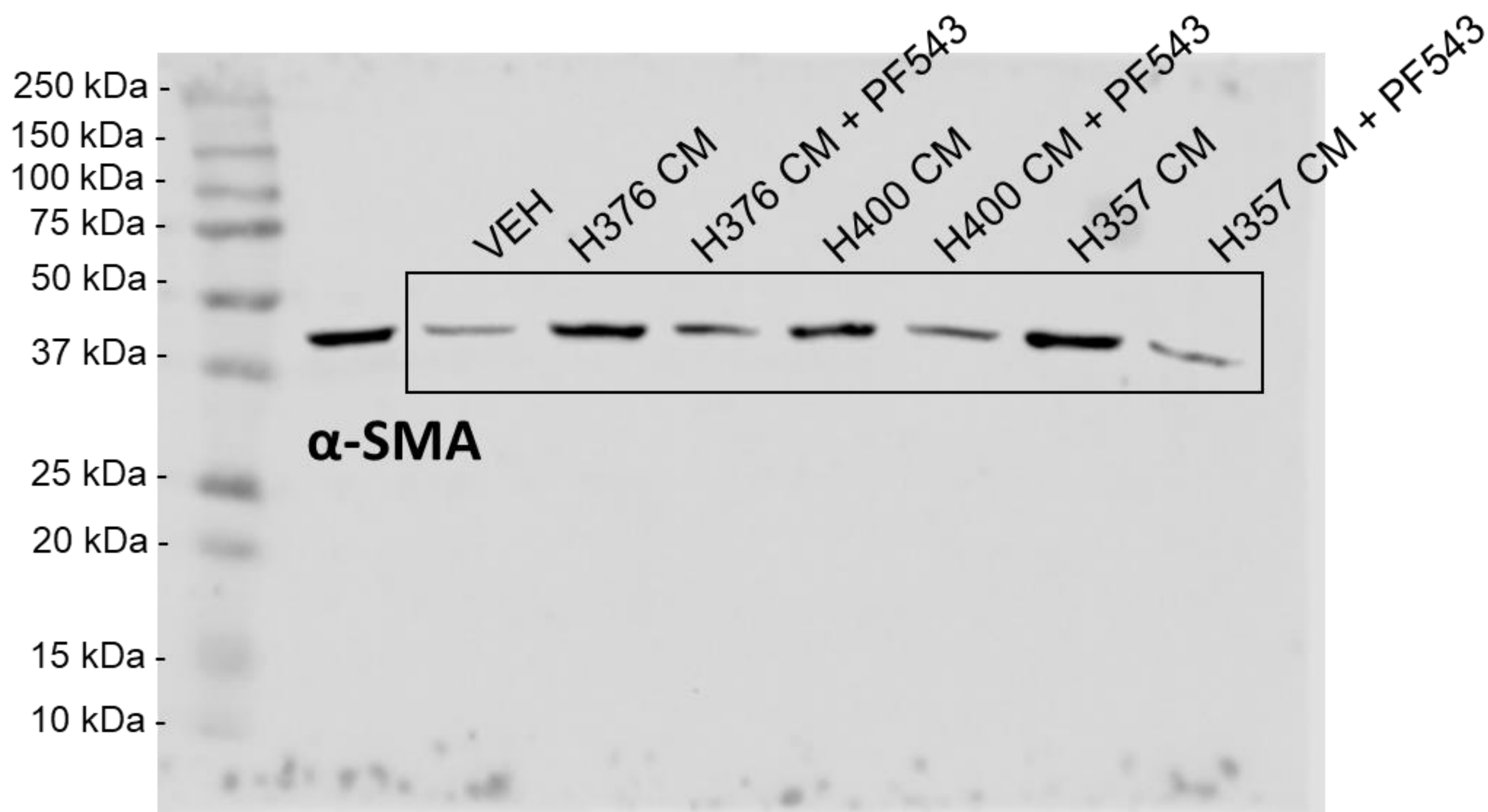

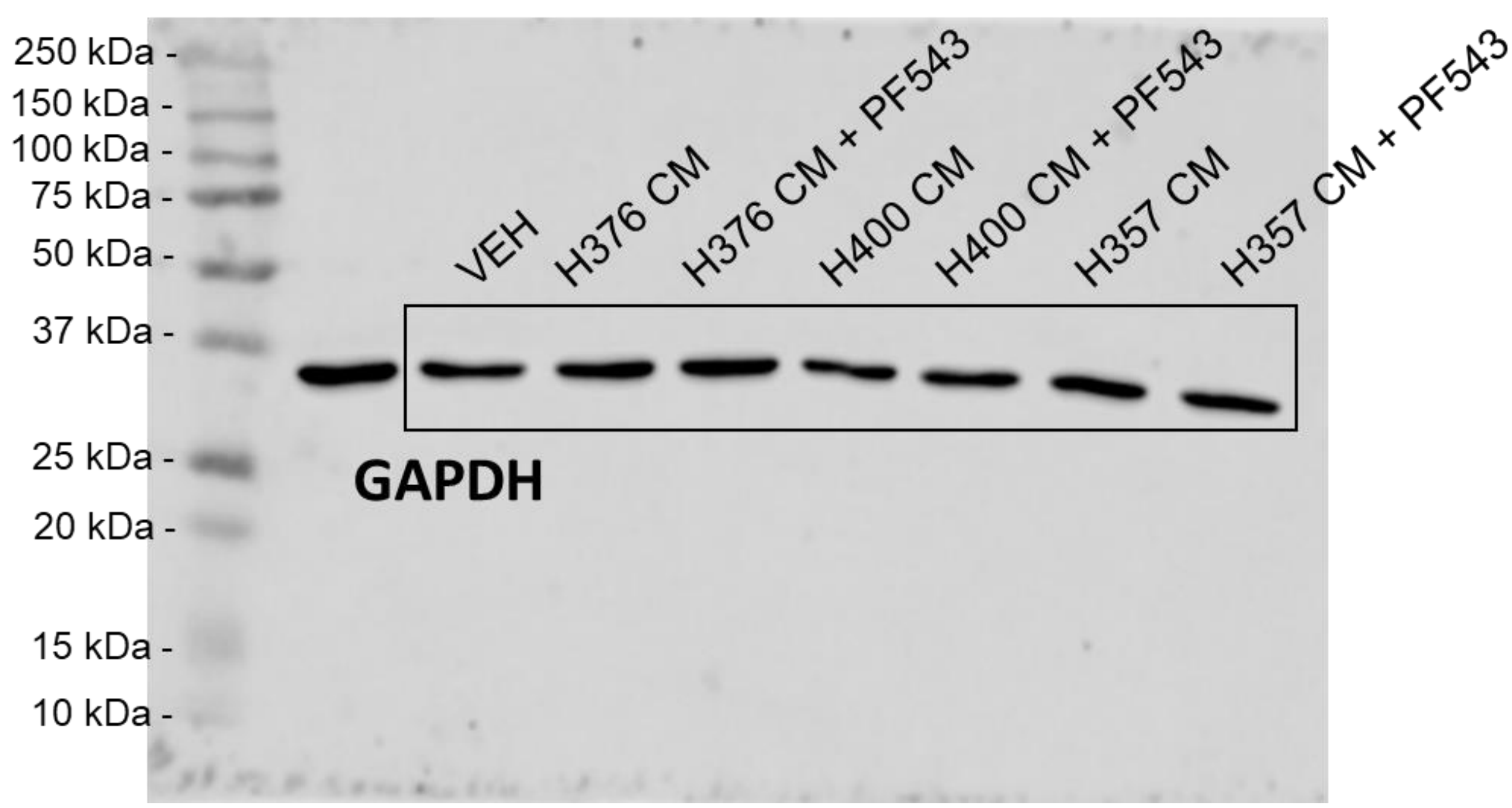

Uncropped blots of Figure 3B

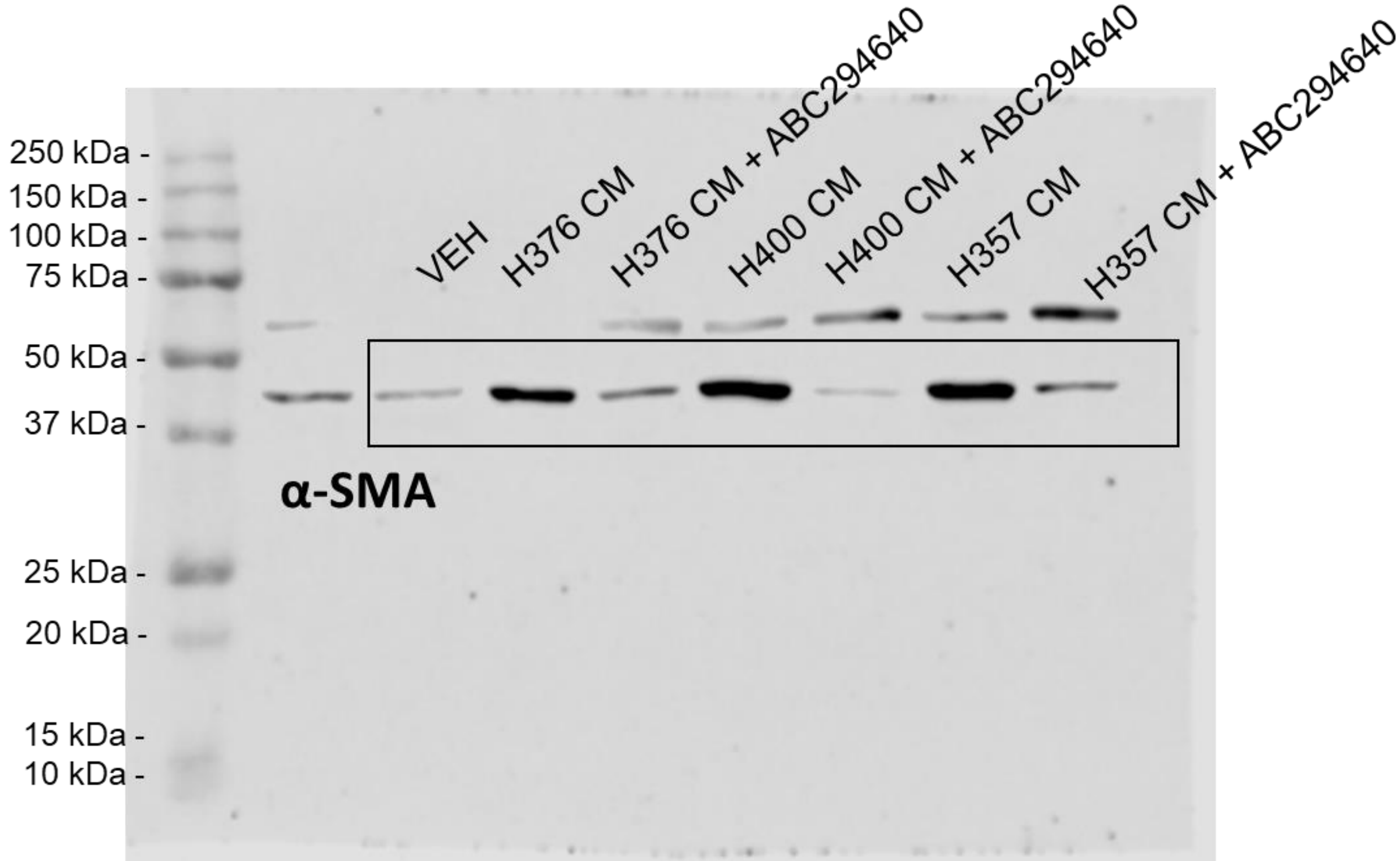

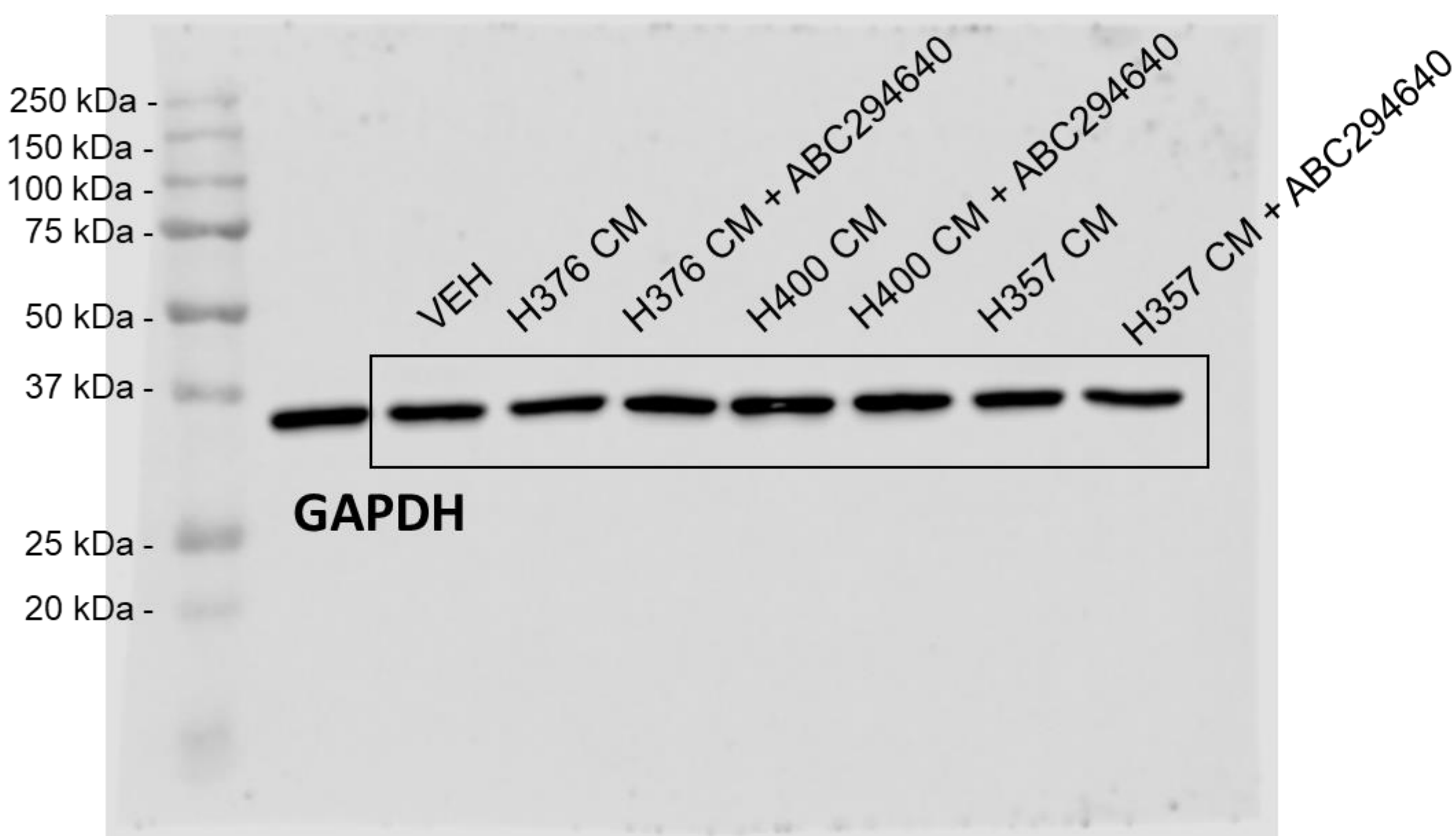

Uncropped blots of Figure 4C

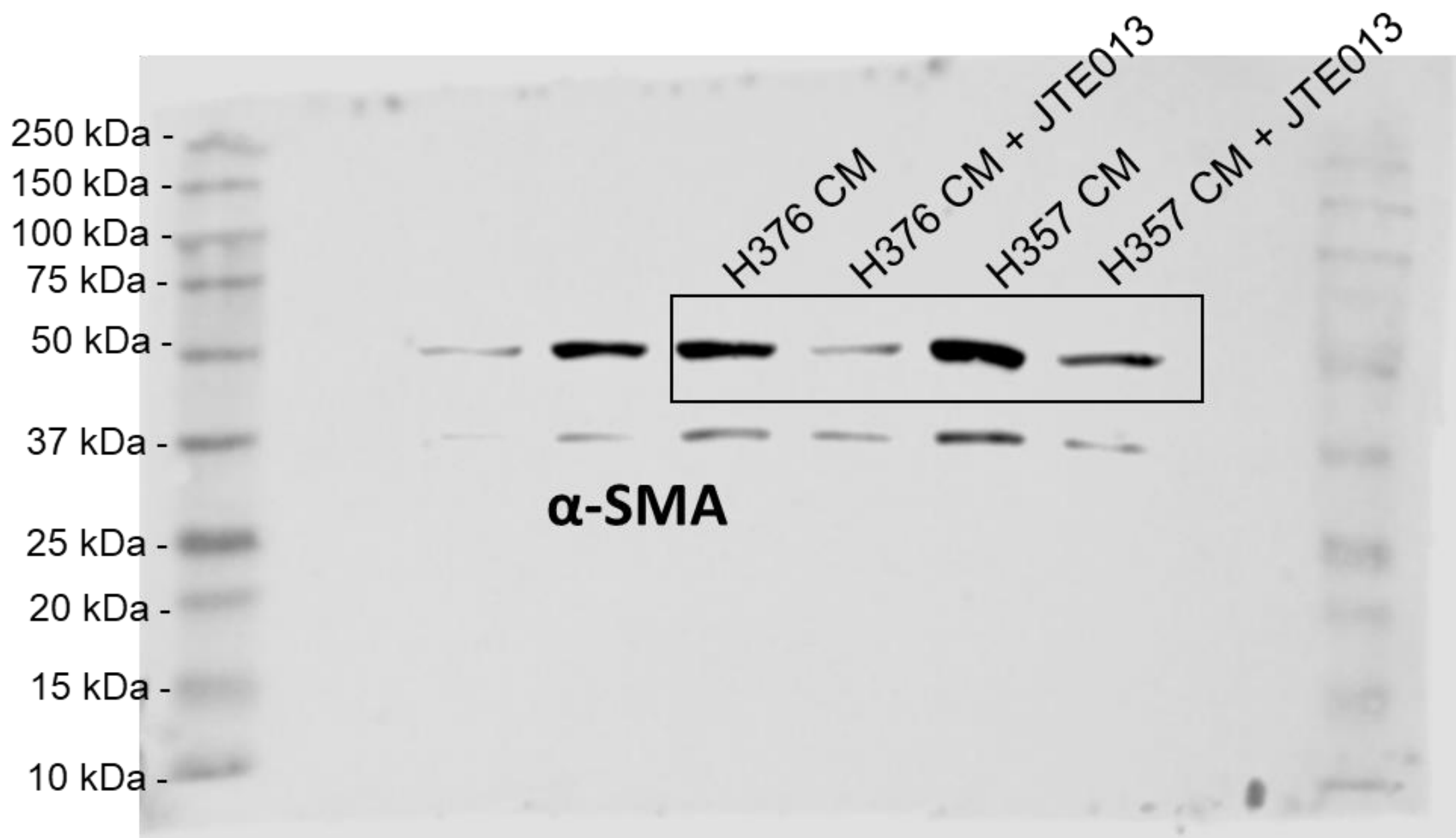

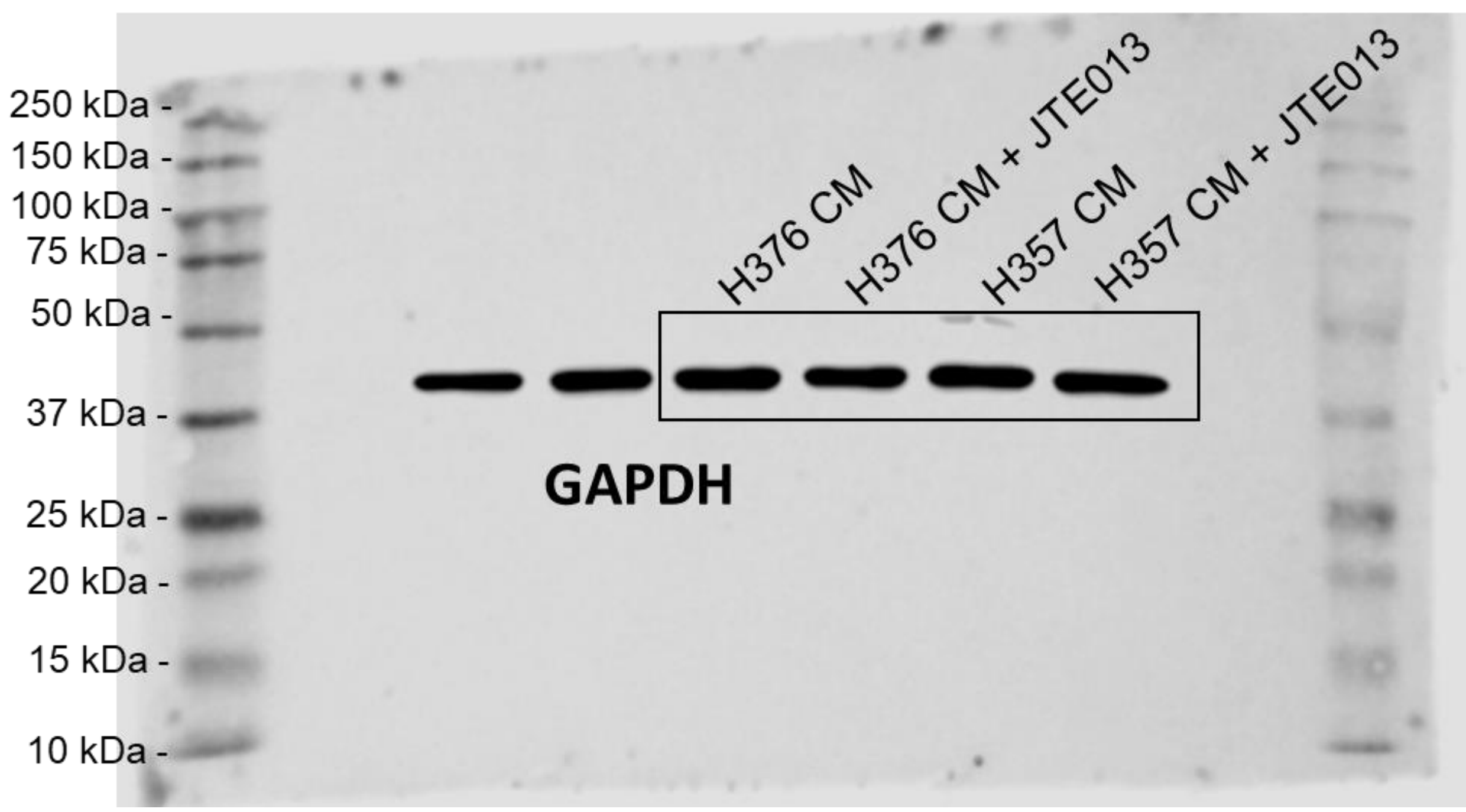

Uncropped blots of Figure 4D

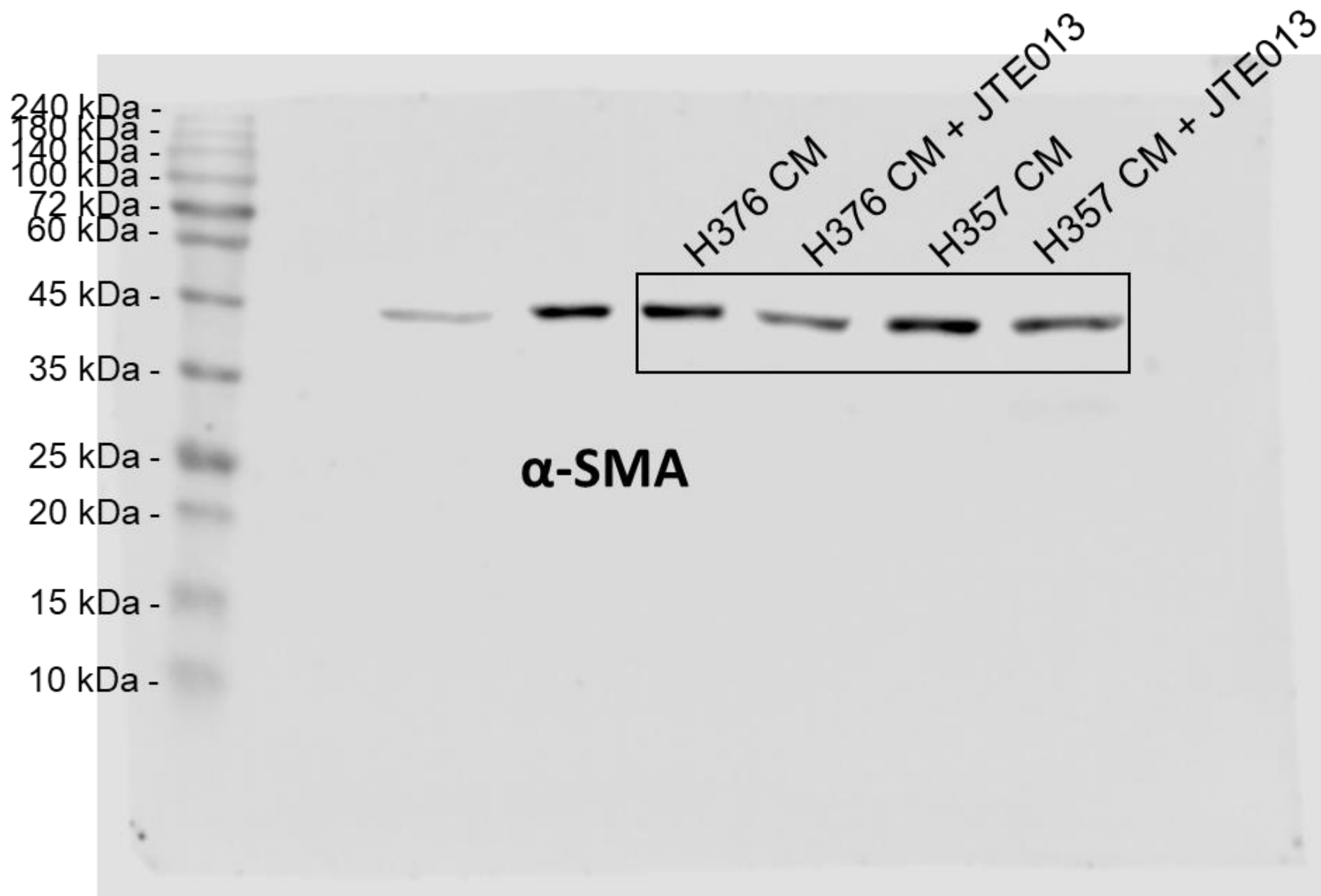

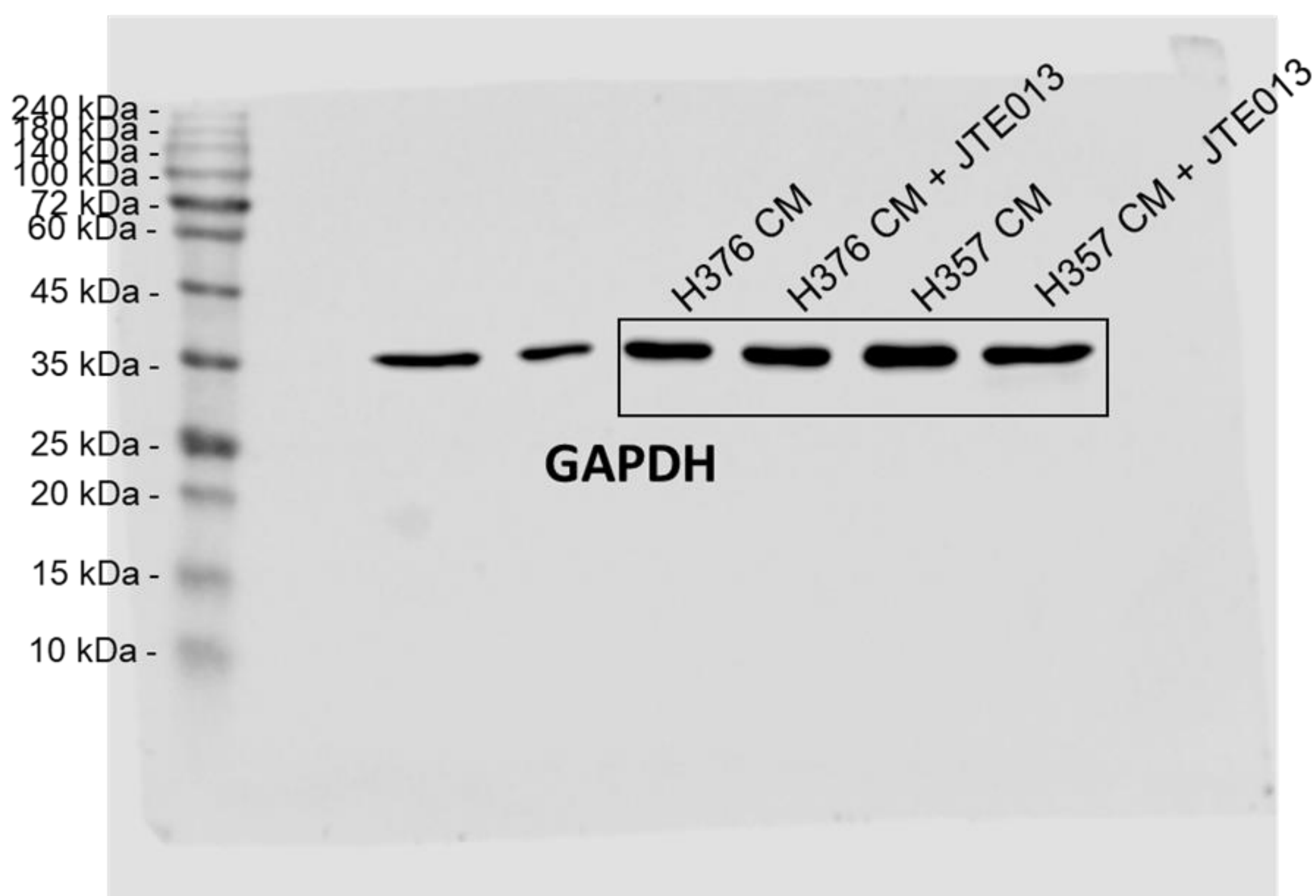

Uncropped blots of Figure 4E

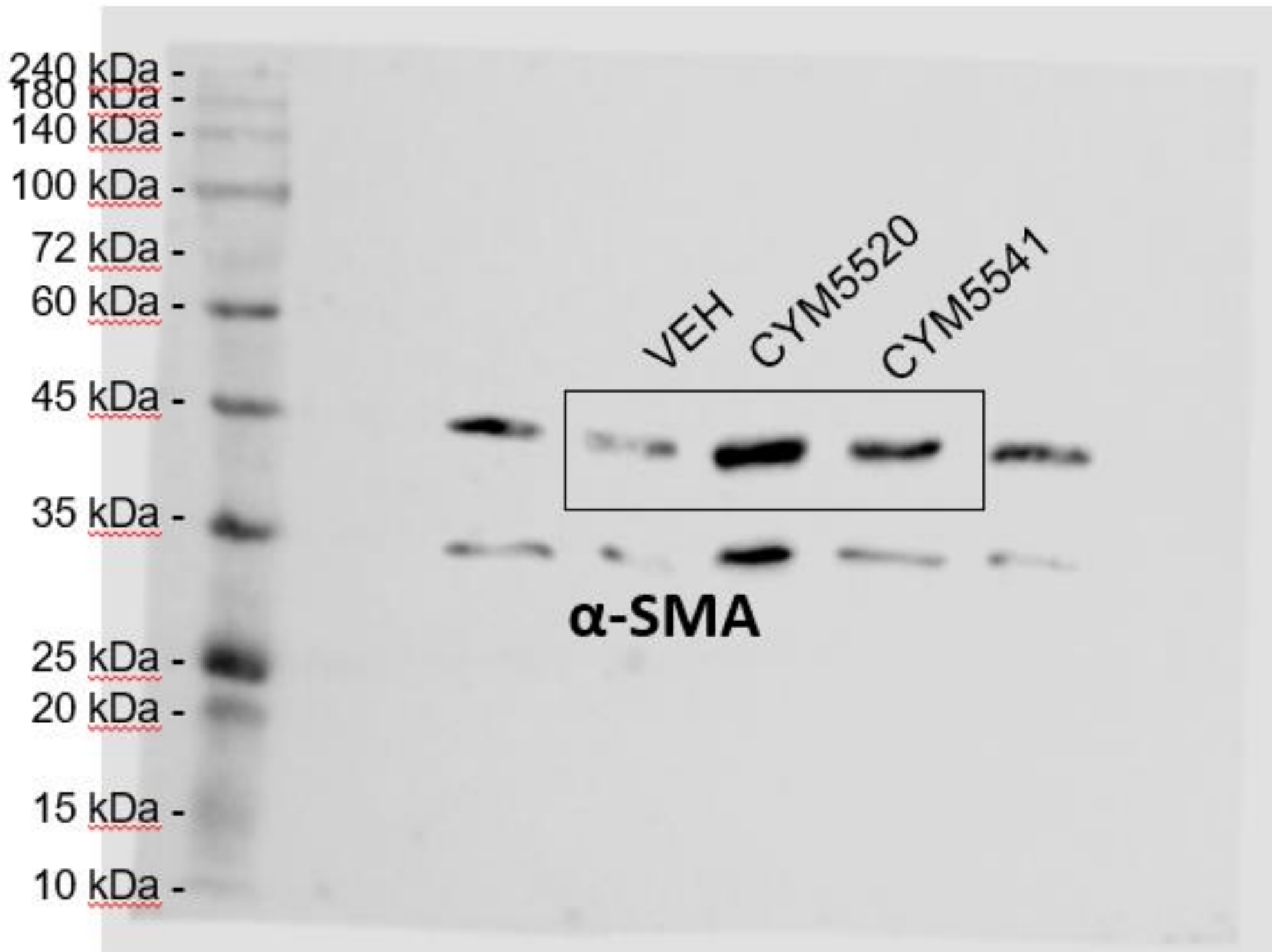

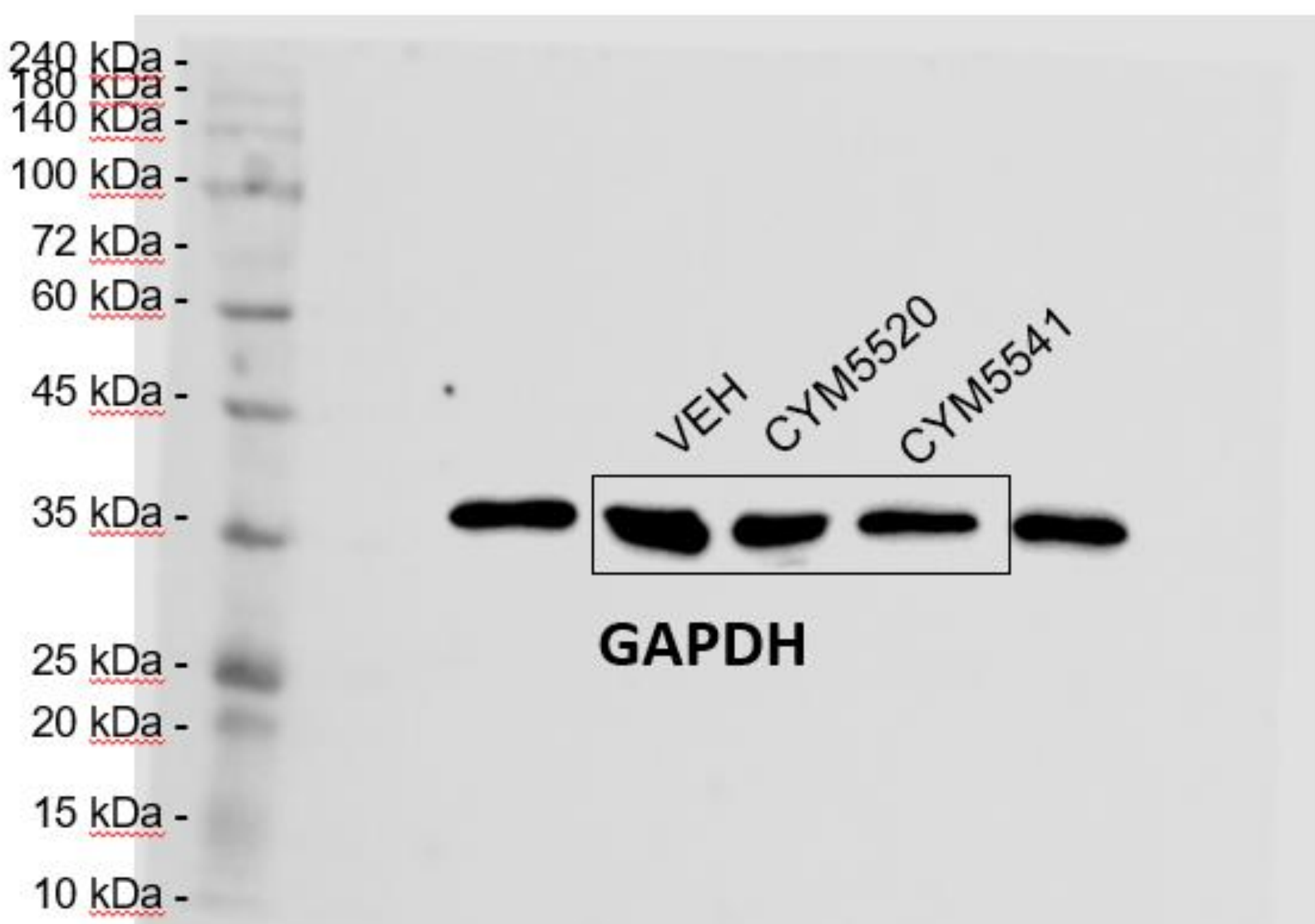

Uncropped blots of Figure 4F

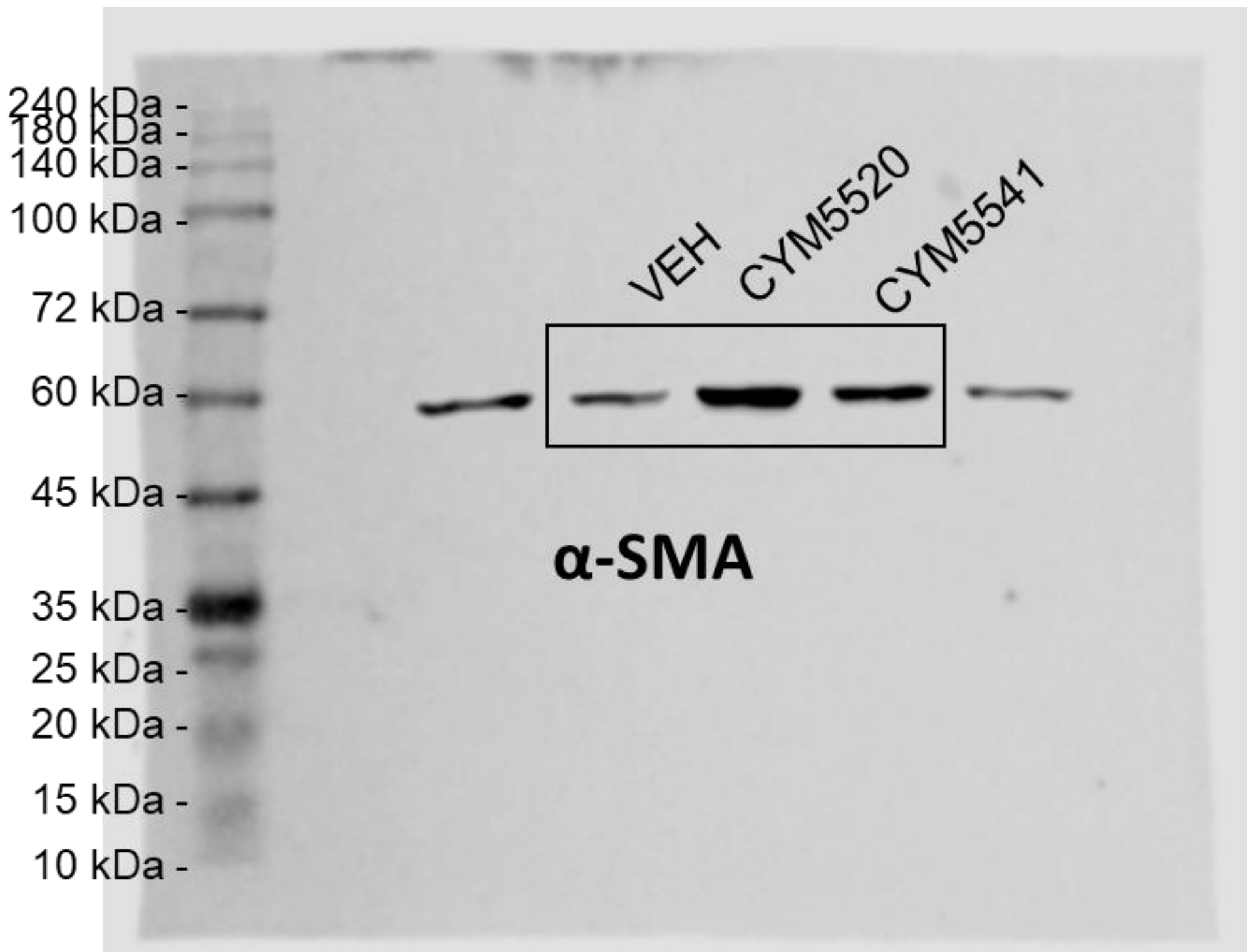

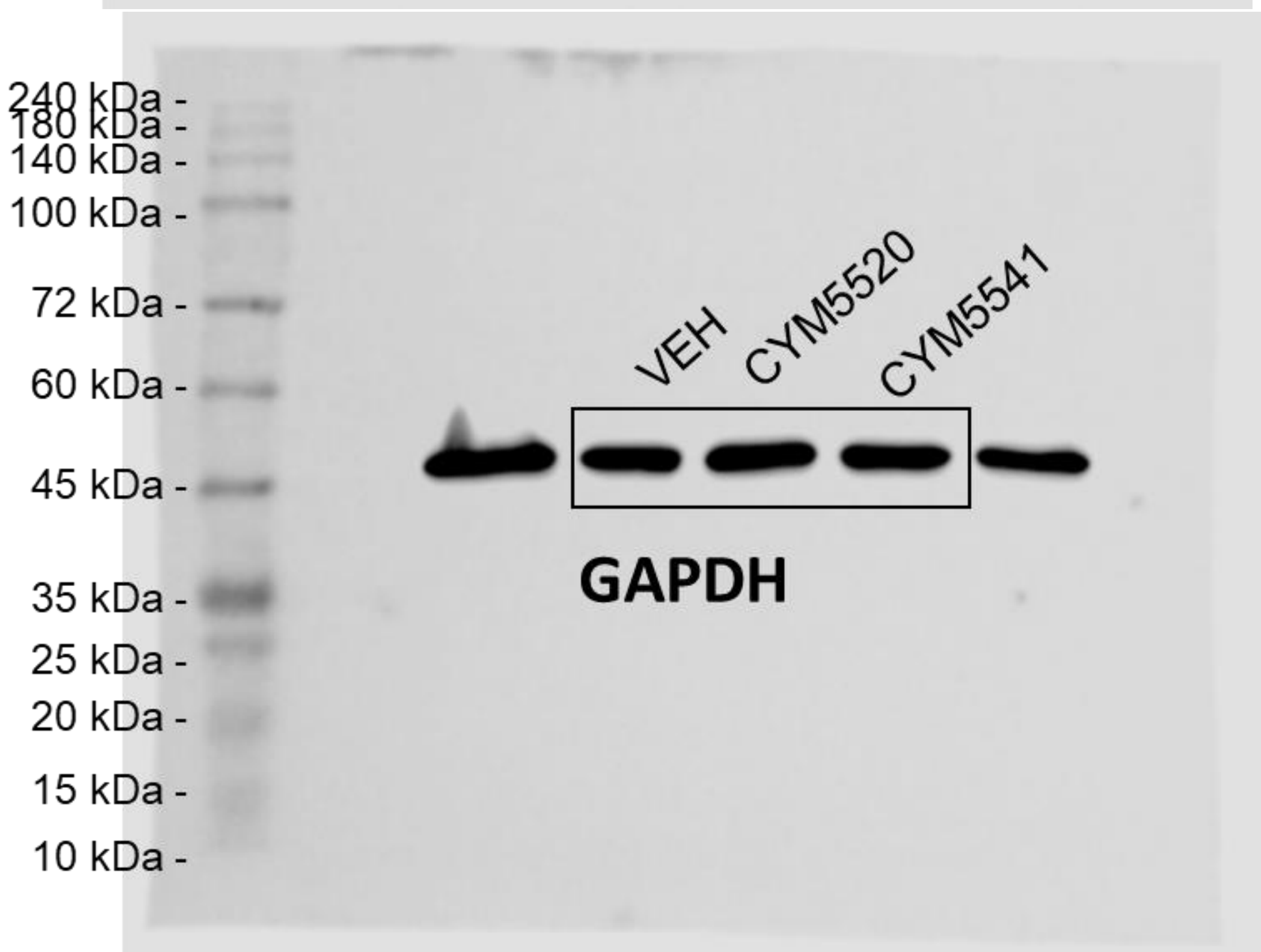

Supplement: Supplementary file 2 [file peerj-14-21673-s002.pdf]
